# Altered Erythro-Myeloid Progenitor Cells Are Highly Expanded in Intensively Regenerating Hematopoiesis

**DOI:** 10.3389/fcell.2020.00098

**Published:** 2020-02-25

**Authors:** Kateřina Faltusová, Chia-Ling Chen, Tomáš Heizer, Martin Báječný, Katarina Szikszai, Petr Páral, Filipp Savvulidi, Nicol Renešová, Emanuel Nečas

**Affiliations:** ^1^Institute of Pathological Physiology, First Faculty of Medicine, Charles University, Prague, Czechia; ^2^BIOCEV, First Faculty of Medicine, Charles University, Vestec, Czechia

**Keywords:** regeneration, stem cell, progenitor cell, adult hematopoiesis, embryonic hematopoiesis, bone marrow, ionizing radiation

## Abstract

Regeneration of severely damaged adult tissues is currently only partially understood. Hematopoietic tissue provides a unique opportunity to study tissue regeneration due to its well established steady-state structure and function, easy accessibility, well established research methods, and the well-defined embryonic, fetal, and adult stages of development. Embryonic/fetal liver hematopoiesis and adult hematopoiesis recovering from damage share the need to expand populations of progenitors and stem cells in parallel with increasing production of mature blood cells. In the present study, we analyzed adult hematopoiesis in mice subjected to a submyeloablative dose (6 Gy) of gamma radiation and targeted the period of regeneration characterized by massive production of mature blood cells along with ongoing expansion of immature hematopoietic cells. We uncovered significantly expanded populations of developmentally advanced erythroid and myeloid progenitors with significantly altered immunophenotype. Their population expansion does not require erythropoietin stimulation but requires the SCF/c-Kit receptor signaling. Regenerating hematopoiesis significantly differs from the expanding hematopoiesis in the fetal liver but we find some similarities between the regenerating hematopoiesis and the early embryonic definitive hematopoiesis. These are in (1) the concomitant population expansion of myeloid progenitors and increasing production of myeloid blood cells (2) performing these tasks despite the severely reduced transplantation capacity of the hematopoietic tissues, and (3) the expression of CD16/32 in most progenitors. Our data thus provide a novel insight into tissue regeneration by suggesting that cells other than stem cells and multipotent progenitors can be of fundamental importance for the rapid recovery of tissue function.

## Introduction

Tissue regeneration is a complex and highly orchestrated process leading to tissue reconstitution and recovery of its function. The hematopoietic system provides a unique opportunity for studying the process of its regeneration due to its easy access and the availability of sophisticated analytical methods including flow cytometry, cell sorting, *in vitro* clonogenic cultures, transplantation assays, and gene expression profiling.

Decades of research into the adult murine hematopoiesis have established a hierarchical organization of hematopoiesis in which hematopoietic stem cells (HSCs) give rise to multipotent progenitors (MPPs), and MPPs further develop into lineage-committed and progressively developmentally restricted progenitor cells which finally give rise to differentiated myeloid and lymphoid precursor cells (Weissman, 2000; Adolfsson et al., 2001; Na Nakorn et al., 2002; Kiel et al., 2005; Yang et al., 2005; Pronk et al., 2007; Wilson et al., 2007; Morita et al., 2010; Oguro et al., 2013).

However, several experimental findings have indicated a more complex organization of the immature hematopoietic cells and also challenged the idea that the extensive self-renewal capacity is a unique property of HSCs (Adolfsson et al., 2005; England et al., 2011; Yamamoto et al., 2013; Kim et al., 2015). It was also demonstrated that the undisturbed murine hematopoiesis is maintained by multiple clones acting in parallel (Zavidij et al., 2012; Sun et al., 2014) without any significant contribution from HSCs. Busch et al. (2015) also demonstrated that undisturbed adult hematopoiesis is largely sustained by cells downstream of HSCs, and Schoedel et al. (2016) reported a long-term hematopoiesis occurring in the absence of HSCs while, in contrast, Sawai et al. (2016) and Akinduro et al. (2018) presented the data supporting the continuous contribution of HSCs for steady state hematopoiesis. The controversy in published reports and the question whether transplantable HSCs are required for adult hematopoiesis have been recently discussed by McRae et al. (2019). Further, the megakaryocyte-deficient lympho-erythro-myeloid progenitors and megakaryocyte-restricted progenitors with the properties of long-term HSC were also described in unperturbed adult hematopoiesis (Carrelha et al., 2018; Rodriguez-Fraticelli et al., 2018).

The formation of adult steady state hematopoiesis wherein HSCs and progenitors steadily generate mature blood cells with limited life-span is preceded by its prenatal and early postnatal expansion derived from a small number of founder cells. During the embryonic, fetal and early postnatal periods of life, hematopoietic tissue has to establish its hierarchical organization in parallel with the essential production of functional blood cells. This represents a non-steady state situation when two contradictory processes co-exist, the one requiring self-renewal of produced cells, while the other one requiring their efficient differentiation, both in competition with each other.

In the mouse, the transient primitive hematopoiesis is established in the yolk sac at the embryonic day E7.5 producing mainly primitive red blood cells which undergo the process of maturation in the circulation. These primitive red blood cells are distinguishable from the later fetal and adult definitive red blood cells by their large size and embryonic globin expression (Palis, 2014). This is followed by emergence of the erythro-myeloid progenitors (EMP), also in the yolk sac, which colonize the fetal liver at E10.5 and give rise to definitive erythrocytes. EMPs also have potential for production of myeloid cells and megakaryocytes but not lymphocytes (Frame et al., 2013; McGrath et al., 2015). These cells lack the capacity to be transplanted and to reconstitute damaged hematopoiesis which is the hallmark of HSCs. The HSCs differentiate later from a specialized hemogenic endothelium in large arteries in the AGM (aorta-gonad-mesonephros) region of the embryo and in the vitelline arteries and also in the placenta [reviewed in Palis (2016); Dzierzak and Bigas (2018)] and are the founder cells for the hierarchically organized adult hematopoiesis producing myeloid and lymphoid blood cells.

A similar situation to that in the embryo/fetus arises in the adult hematopoiesis after a severe bone marrow damage. The hierarchy of immature hematopoietic cells has to be reconstituted in parallel with the life-saving recovery of blood cell production. McCarthy (1997) demonstrated that after irradiation of mice with a dose of 6 Gy some mice develop, after several months, monoclonal hematopoiesis derived from a single cell. This demonstrates that in the adult life, after such a significant damage to the bone marrow, hematopoiesis can finally re-establish its HSCs-progenitor cells hierarchy. However, a detailed knowledge about the period of early regeneration of adult bone marrow when it expands the populations of immature cells together with intensive production of blood cells is lacking. We attempt here to fill this significant gap in knowledge by comprehensively examining the immature lineage negative and c-Kit positive hematopoietic cells (Lin^–^c-Kit^+^; LK cells) and their subsets in bone marrow at its vigorous regeneration phase following its major damage induced by total body irradiation of mice with a dose of 6 Gy. We describe here the significant alterations of the immunophenotype, the developmental potential and programing, and the capacity for transplantation of immature hematopoietic cells in regenerating bone marrow. We show that the regenerating hematopoietic system significantly differs from the expanding hematopoiesis in the fetal liver and early postnatal bone marrow, but find some similarities with the early embryonic definitive hematopoiesis driven by myeloid progenitors before the emergence of transplantable HSCs.

## Materials and Methods

### Mice

C57BL/6J (CD45.2) and B6.SJL-Ptprc^a^ Pepc^b^/BoyJ (CD45.1) mice were bred in a specific-pathogen free facility of the Center for Experimental Biomodels, First Faculty of Medicine, Charles University and housed in a clean conventional part of the facility (12:12 h light-dark cycle, 22 ± 1°C, 60 ± 5% humidity) during the experiments. Adult mice (mostly 8–12 weeks old) of both sexes were used in the experiments. To gain mice fetuses of the required developmental stage, female mice in estrus were placed in cages with males (two females with one male). The females were checked in the morning and those with the copulation plug were followed for the pregnancy starting at the E0.5 day. All experiments were performed in accordance with national and international guidelines for laboratory animal care and approved by the Laboratory Animal Care and Use Committee of the First Faculty of Medicine, Charles University and the Ministry of Education, Youth and Sports of the Czech Republic (MSMT-6316/2014-46 and MSMT-4502/2017-2).

### Irradiation

Irradiation was performed in a plastic cage and a ^60^Co source (Chisobalt 2-B75, MEDIN, formerly Chirana, Czechia) from a distance of 123.5 cm with a dose rate of ∼0.35 Gy per minute.

### Spleen Colonies (CFU-S)

Spleen colonies were determined in submyeloablatively irradiated mice (4 Gy or 6 Gy) 8–14 days after irradiation (endogenous spleen colonies). Spleens were fixed in Tellesniczky′s solution (18:1:1 volume parts of 70% ethanol, glacial acetic acid and formalin) and spleen colonies were observed with the naked eye and counted.

### Bone Marrow Collection

Bone marrow cells were obtained from the long bones (femurs and tibias, or femurs only) by flushing the bone cavity with PBS supplemented with 1% bovine serum albumin (BSA) through a hole in one end of the bone without clipping off the epiphyses. A single-cell suspension was obtained by repeated passage through the needle (25G) and kept on ice before further handling.

### Flow Cytometry and Cell Sorting

Cells were filtered through a 70 μm nylon cell filter (BD Biosciences, San Jose, CA, United States) and stained with fluorochrome-labeled antibodies. Bone marrow cell populations were defined by immunophenotyping and forward (FSC) and side (SSC) scatter characteristics. Bone marrow cells were stained by fluorescently labeled antibodies for 20 min at 4°C in the dark with optimal dilutions of commercially prepared antibodies listed in Supplementary Table S1.

Stained bone marrow cells were analyzed using a digital FACS Canto II flow cytometer, equipped with 405 nm (60 mW), 488 nm (20 mW), and 633 nm (15 mW) lasers and the relevant configuration of optical filters and signal detectors (BD Biosciences). For data acquisition, BD FACSDiva software version 6.1.3 was used. CS&T beads (BD Biosciences) were used for the automated cytometer setup and the performance tracking procedure before measurements. A compensation matrix was created by running single-stained control samples (automatic compensation). The compensation matrix was then controlled and manually adjusted (if necessary) in each measurement. The generated flow cytometry data were analyzed using FlowJo vX software (FlowJo, Tree Star, OR, United States). Debris, red blood cells and dead cells were excluded from the analysis by gating the FSC-A/SSC-A dot plot. For cell doublet discrimination, a FSC-A/FSC-H dot plot was used. To properly interpret flow cytometry data, Fluorescence-Minus-One (FMOs) controls were used for gating. Recordings containing less than 50 cells of a particular phenotype were excluded from further analysis.

Cells were sorted with a FACSAria IIu cell sorter (BD Biosciences) equipped with 489 nm (50 mW), 561 nm (100 mW), 638 nm (140 mW), 404 nm (100 Mw), and 355 nm (20 mW) lasers. Cells were sorted with the use of either 70-micron or 85-micron integrated nozzle (with corresponding sheath pressure), under a “0-18-0” precision mode setup (yield mask 0, purity mask 18, phase mask 0). A compensation matrix was created by running single-stained control samples (automatic compensation). The compensation matrix was then checked and manually adjusted (if necessary) before each sorting procedure. Cells were sorted into polypropylene microcentrifuge tubes (Eppendorf, Hamburg, Germany) containing 1x PBS supplied with 3–5% albumin fraction V, biotin-free (Carl Roth GmbH, Karlsruhe, Germany). Before cell sorting, “Checking cytometer performance” (CS&T) and “Determining the drop delay” (BD Biosciences Accudrop beads) procedures were executed. Sterile 1x PBS was used as sheath fluid. BD FACS Diva software version 6.1.2 was used for data acquisition.

### Identification of Immature Lineage Negative c-Kit Positive (LK) Cells and Their Subsets

LK cells and their subsets were identified by flow cytometry as is shown in result figures. LK cells were analyzed in their c-Kit^high^ and c-Kit^low^ fractions.

### Red Blood Cell Transfusion

Mice under deep anesthesia were exsanguinated from the retro-orbital venous sinus by heparinized capillaries and sacrificed by cervical dislocation. Red blood cells were washed with an excess of PBS and a 75% suspension of red blood cells in PBS was prepared. The suspension was intravenously injected in a volume of 0.5–0.6 ml to mice via the retro-orbital route (29G needle).

### Erythropoietin Given to Normal Mice

Male mice were injected with recombinant human erythropoietin (NeoRecormon epoetin beta; Roche, Basel, Switzerland; EPO) intraperitoneally for four consecutive days with a cumulative dose of 200 IU/mouse. Bone marrow was collected 24 h after the last EPO injection.

### A State of Iron Deficiency

Male mice were put on a low-Fe diet (C 1038; Altromin Spezialfutter GmbH & Co., KG, Hamburg, Germany) for 7 days, and were bled 0.5–0.6 ml from the retro-orbital venus sinus 5 days and 1 day before the examination of peripheral blood and bone marrow.

### ACK2 c-Kit Blocking Antibody Administration

ACK2 *In Vivo* Ready^TM^ Anti-Mouse CD117 (c-Kit) was used as the c-Kit blocking antibody. The reagent was from Tonbo Biosciences (United States). ACK2 antibody was administered to mice intravenously (0.5 mg in 0.25 ml per mouse).

### Peripheral Blood Analysis

Peripheral blood was collected from the retro-orbital venous sinus of anesthetized mice using capillaries (75 mm/60 μl; KERAGLASS, Otovice, Czechia) containing a small volume of EDTA and was analyzed with a BC-5300Vet Auto Hemato Analyzer (Mindray Bio-Medical Electronics, Shenzhen, China) calibrated for mouse blood samples.

### *In vitro* Cultivation of Bone Marrow Cells in Semi-Solid Media

Bone marrow cells (pooled from 3 mice) of either untreated mice or mice irradiated (6 Gy) 14 days prior were cultured in duplicates on 30-mm Petri dishes in 1 ml of one of three types of MethoCult^TM^ semi-solid media (STEMCELL Technologies, Vancouver, BC, Canada). 3 × 10^4^ (GF M3434 medium), 1 × 10^5^ (SF M3436 medium) or 2 × 10^5^ (M3334 medium) cells of normal bone marrow were plated per dish. Sorted LSK and LS^neg^K cells were plated in 200–1000 numbers per dish. Single cells were sorted into a 96-well plates for the analysis of cells with different CD71 expression level. The cultures were conducted at 37°C in a humidified air atmosphere with 5% CO_2_ for 2 days in the M3334 medium (CFU-E clusters) and for 8–12 days in the SF M3436 and GF M3434 media. Colonies were counted and analyzed by phase-contrast light microscopy and evaluated according to STEMCELL Technologies’ Mouse Colony-Forming Unit (CFU) Assays Technical Manual (v 3.2.0; Document # 28405). Examples of the CFU-E clusters and BFU-E, CFU-GEMM, CFU-GM, CFU-G, CFU-M colonies are shown in Supplementary Figure S1.

In three independent experiments, either LSK or LS^neg^K were sorted from bone marrow pooled from three normal or three mice irradiated 14 days before bone marrow collection. 200 LSK cells or 500 LS^neg^K cells sorted from the normal bone marrow and twice as many cells sorted from the regenerating bone marrow were cultured in GF M3434 medium per dish. Colonies were classified according to their type into CFU-GEMM, CFU-M, CFU-G, CFU-GM or BFU-E.

### Reverse Transcription Quantitative PCR

RNA was isolated from samples of whole bone marrow, bone marrow depleted of lineage positive cells by magnetic cell sorting (MACS; Miltenyi Biotec), or sorted Sca-1^negative^ and Sca-1^positive^ LK cells (LS^neg^K and LSK cells). The mouse lineage cell depletion kit (MACS; Miltenyi Biotec) was used according to the manufacturer’s protocol. Briefly, bone marrow cells were labeled with a cocktail of biotin-conjugated monoclonal antibodies against lineage markers CD5, CD45R (B220), CD11b, Gr-1 (Ly-6G/C), 7-4 and Ter-119 and were labeled with anti-biotin monoclonal antibodies conjugated to superparamagnetic beads. The magnetically labeled cells were loaded into MACS LS columns and lineage positive cells were removed from bone marrow with the use of a strong permanent magnetic field. The efficiency of lineage depletion by magnetic cell sorting was confirmed by flow cytometric analysis of magnetically depleted samples (not shown).

Total RNA was isolated using RNeasy Plus Mini kit, RNeasy Plus Micro kit, or QIAzol Lysis Reagent (all from QIAGEN). RNA quantitative and qualitative analyses employed Agilent RNA 6000 Pico Kit (Agilent Technologies). An iScript^TM^ cDNA Synthesis kit (BIO-RAD) was used for cDNA synthesis.

RT-qPCR was performed using a 7900HT Fast Real-Time Cycler (Applied Biosystems) and 2xSYBR Green qPCR Master Mix (Bimake). Each sample was done in triplicate, CT values from triplicates were calculated as mean CT, the results were expressed as relative gene expression by 2^–ΔΔ^ ^CT^ (Figure 4B, Supplementary Figure S5B) or 2^–Δ^ ^CT^ (Supplementary Figure S5A) methods. Each sample was normalized to the level of mRNA of housekeeping genes. Primer sequences are listed in Supplementary Table S2.

### Bone Marrow Transplantation

A single cell suspension of bone marrow cells was transplanted intravenously through the retro-orbital route. Recipient mice were dual CD45.1/CD45.2 (F1) mice irradiated at 8.5 Gy prior to the transplantation. The recipients were transplanted with a mixture of bone marrow cells or sorted LSK CD150^+^CD48^–^ cells of regenerating (CD45.2) and untreated (CD45.1) mice (*vice versa* in one experiment; Table 2). Chimeric CD45.2/CD45.1 bone marrow from transplanted mice was retransplanted to secondary 8.5 Gy-irradiated F1 recipient mice. The experimental design of the transplantation assays is shown in Figure 6D.

### Analysis of Chimeric Hematopoiesis in Peripheral Blood

The ratio of donor to host nucleated blood cells was determined in samples of peripheral blood drawn from the retro-orbital venous plexus of transplanted mice using capillaries containing 5 μL of 0.5M EDTA. Approximately 50 μL blood samples were stained with anti-CD45.1 and anti-CD45.2 antibodies for 30 min on ice in the dark and washed after. The samples were also stained for Gr-1/Mac-1, B220, and CD3 or CD4 and CD8 markers. Only CD45.1 or CD45.2 single-positive cells were evaluated by flow cytometry, the double CD45.2/CD45.1-positive cells were excluded from analysis.

### Analysis of Chimeric Hematopoiesis in Bone Marrow

Femoral bone marrow cells were collected into PBS. To determine the ratio between CD45.2 cells (originating from regenerating bone marrow) and CD45.1 cells (originating from control bone marrow; *vice versa* in one experiment), four million of bone marrow cells were washed with PBS and centrifuged (4°C, 400 *g*, 6 min). After removing supernatant, the cells were stained for the CD45.2 and CD45.1 allotypes and also for Gr-1/Mac-1, B220 and CD3 or CD4 and CD8 markers. Another set of samples were stained for CD45.2 and CD45.1 markers and with lineage cocktail, anti-c-Kit and anti-Sca-1 antibodies.

### Statistical Methods

Mostly multiple independent experiments were performed to verify the reproducibility of all experimental findings. Statistical analysis was done with GraphPad Prism version 5 (GraphPad Software, La Jolla, CA, United States). Two-tailed unpaired Student′s *t*-tests were used to determine statistical significance when two groups were compared. One-way analysis of variance (ANOVA) using Dunnett′s post-test was used to compare each group to the control group when more than two groups were evaluated. Results are presented as means ± SEM or ± SD in Tables 1–3.

**TABLE 1:** Number of spleen colonies in submyeloablatively irradiated mice.

Day	8	11*	12*	13*	14*
4 Gy	12.5 ± 1.9 (4)	n.d.	n.d.	n.d.	n.d.
6 Gy	0.6 ± 0.3	0.4 ± 0.2	0.2 ± 0.2 (^#^)	0.6 ± 0.2 ^#^	0.3 ± 0.2 ^#^
	(12)	(14)	(6)	(24)	(24)

Values are means ± SD; number of spleens is indicated in brackets; * – colonies > 2mm; ^#^ – large number of very small colonies; n.d. – non-determined.

**TABLE 2:** Chimeric hematopoiesis resulting from co-transplantation of bone marrow cells from normal or irradiated mice containing equal number of LSK cells; two independent experiments (6 Gy 14D and 6 Gy 23D).

Experiment	PB examined after	F1 (recipients)	Normal BM (CD 45.1)	Regenerating BM (CD 45.2)	45.1/45.2 ratio
6 Gy 14D	1 month	38.63 ± 6.33	60.63 ± 6.34	0.74 ± 0.09	83
	6 months	33.76 ± 6.62	66.1 ± 6.60	0.15 ± 0.05	478
6 Gy 23D	1 month	21.28 ± 3.82	77.55 ± 3.77	1.16 ± 0.29	70
	6 months	12.34 ± 4.39	87.41 ± 4.34	0.25 ± 0.12	516

*Five dual CD45.1/CD45.2 F1 (F1) hybrid mice were irradiated (5 Gy) and transplanted with a mixture of bone marrow (BM) cells from normal (CD45.1) and (CD45.2) irradiated mice. Mice were irradiated either 14 days (14D) or 23 (32D) prior to BM collection. The number of LSK cells was determined in pooled samples of the normal and the regenerating BM cells by flow cytometry and the two BM samples were mixed in such a ratio that 10,000 CD45.1 and 10,000 CD45.2 LSK cells were administered to F1 recipient mice. The transplanted cells competed with each other and against the repopulating cells which survived in submyeloablatively irradiated F1 recipient mice. The contribution of the three types of repopulating cells to blood cell production was examined in the peripheral blood (PB) of F1 transplant recipients after 1 and 6 months and their proportion is shown as mean ± standard deviation. PB – peripheral blood; BM – bone marrow; D – day after irradiation of mice at 6 Gy.*

**TABLE 3:** Chimeric hematopoiesis derived from LSK CD150^+^CD48^–^ cells sorted from normal and regenerating bone marrow.

Examined after	F1 recipients	LSK CD150^+^48^–^cells from normal BM (CD 45.2)	LSK CD150^+^48^–^cells from regenerating BM (CD 45.1)	45.2/45.1 ratio
1 month PB	57.60 ± 7.31	33.59 ± 9.24	8.81 ± 2.95	3.8
4 months PB	25.58 ± 13.82	70.21 ± 11.65	2.21 ± 0.66	31.8
5 months BM	33.45 ± 19.10	66.01 ± 19.44	0.54 ± 0.47	123.2

*Five dual CD45.1/CD45.2 F1 (F1) hybrid mice were irradiated (7 Gy) and transplanted with 500 + 500 cells with the LSK CD150^+^CD48^–^ immunophenotype sorted either from normal (CD45.2) or regenerating (CD45.1) bone marrow (BM). The regenerating BM was collected 20 days after irradiation. The transplanted cells competed with each other and against the repopulating cells which survived in F1 recipient mice. The contribution of the three types of repopulating cells to blood cell production was examined in the peripheral blood (PB) of recipient F1 mice after 1 and 4 months, and in BM after 5 months. The percentage of cells with the dual CD45.1/CD45.2 (recipient origin), CD45.2 (normal BM origin) and CD45.1 (regenerating BM origin) immunophenotypes in PB and BM is presented as the mean ± standard deviation. The gating used for sorting of LSK CD150^+^CD48^–^ cells is shown in Supplementary Figure S6. PB – peripheral blood; BM – bone marrow.*

## Results

### Immature Hematopoietic Cells Expand Their Population Rapidly and in Parallel to Increasing Production of Blood Cells in Regenerating Hematopoiesis Following Significant Damage

To define the experimental model of hematopoietic regeneration used in the present study, we estimated the extent of the initial damage inflicted on the hematopoietic tissue by irradiating mice with a dose of 6 Gy by determining the occurrence of endogenous spleen colonies arising from the myeloid progenitor cells which survived irradiation. The colony numbers are shown in Table 1 and examples of spleens are shown in Supplementary Figure S2A. There were only occasional spleen colonies in mice irradiated with a dose of 6 Gy. The rare colonies became large after 11 days and from the kinetics of the spleen colony development (Nečas and Znojil, 1989) it can be deduced that they originated from cells which had survived after irradiation and began their clonal expansion shortly thereafter. Numerous very small colonies became visible on spleens examined 12–14 days after irradiation. We hypothesize that they reflect migration of progenitors from regenerating bone marrow to the spleen as was reported by Peslak et al. (2012). Based on the spleen colony results, as well as on the results from our previous study (Forgacova et al., 2013) and the results reported by McCarthy (1997), we estimate that only very few cells with the hematopoietic tissue reconstituting capacity survived in the 6 Gy-irradiated mice.

The population of immature LK cells rapidly expanded between the 14th and the 15th days after irradiation (Supplementary Figure S2C). An intensive production of blood cell occurred 12 days and later after irradiation as indicated by the increasing number of red blood cells in peripheral blood (Supplementary Figure S2B). Altogether, these results suggest the similarity between the vigorously regenerating adult hematopoiesis and the physiologically expanding embryonic/fetal liver and early postnatal hematopoiesis that lies in the concomitant increase of blood cell production and the expansion of populations of immature hematopoietic cells.

### The Immunophenotype of LK Cells Is Significantly Altered in Regenerating Bone Marrow

**FIGURE 1:**
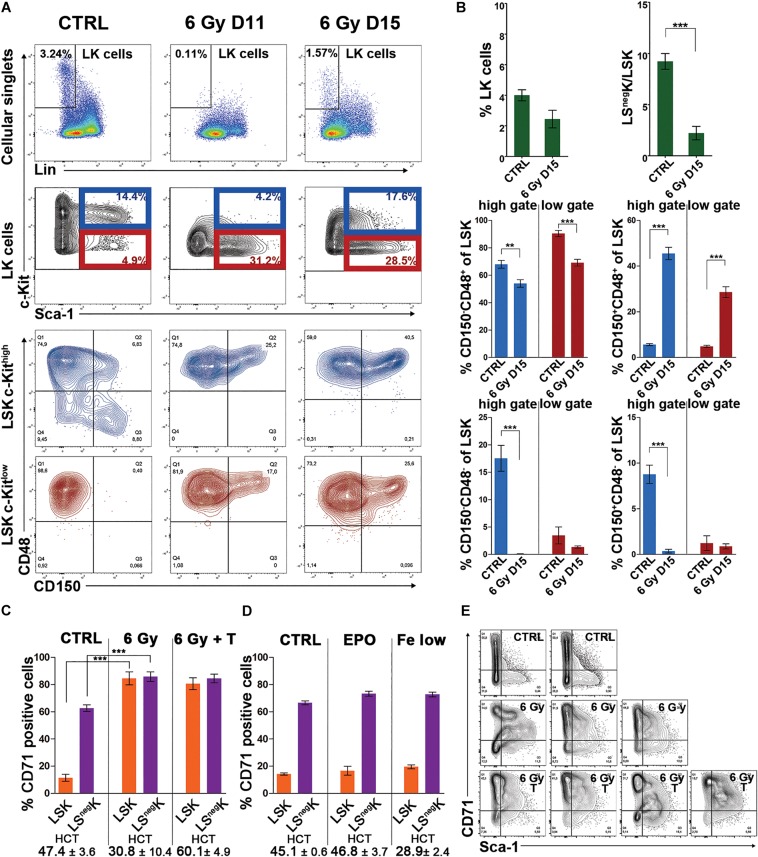
LK cells are c-Kit^low^ and are enriched for Sca-1^+^ cells; LSK cells are CD48^+^, CD71^+^ enriched for CD150^+^ in regenerating bone marrow from irradiated mice. **(A)**, Examples of immunophenotypes of LK cells present in normal bone marrow (CTRL) and bone marrow collected 11 (6 Gy D11) or 15 (6 Gy D15) days after irradiation of mice with 6 Gy. The CD150/CD48 expression profile was determined in c-Kit^high^ and c-Kit^low^ subsets of LSK cells. **(B)** The percentage of LK cells and the ratio between LS^neg^K and LSK cells in normal (CTRL) and regenerating (6 Gy D15) bone marrow. All LK cells were analyzed as shown in **A** by cell dotplots. However, the next analysis of LSK cells regarding their distribution into the four CD150/CD48 expression profile subtypes was made separately for the LSK cells with “high” and “low” c-Kit expression level distinguished by the blue and the dark red colors. Data were pooled from three independent experiments including a total of 5 untreated mice and 8 mice examined 15 days after irradiation. **p < 0.01, ***p < 0.001. **(C)** Percentage of CD71 (transferrin receptor 1) positive LK cells increased in regenerating bone marrow, particularly in LSK cells. All LK cells, as defined in **A**, were divided into LS^neg^K and LSK cells and analyzed. To prevent the development of post-irradiation anemia, in a part of mice, transfusions of red blood cells were started 5 days after irradiation and were given in 3–4 day intervals. Polycythemia induced in irradiated mice by the repeated transfusions of red blood cells did not abolish the increase in CD71 expression. Data were pooled from five independent experiments including a total of 6 untreated mice (CTRL), 13 irradiated mice (6 Gy), and 15 irradiated and transfused mice (6 Gy + T). Mice were examined on day 14 in three experiments and on days 15 and 16 after irradiation in another two experiments. **(D)** Administration of erythropoietin (EPO) to normal (non-irradiated) mice, or an iron-deficient diet combined with bleeding (Fe low), did not influence the expression level of CD71 in LK cells. **(E)** The Sca-1/CD71 expression profile of LK cells, highly conserved in normal bone marrow, became erratic in regenerating bone marrow and this was not abrogated by transfusions of red blood cells. Examples of the Sca-1/CD71 immunophenotypes in LK cells from 2 untreated (CTRL), 3 irradiated (6 Gy) and 4 irradiated and transfused (6 Gy T) mice examined 15 days after irradiation are shown. HCT – hematocrit; T – transfusions of red blood cells.

First, we analyzed the immunophenotype of LK cells in intensively regenerating bone marrow by flow cytometry. We used the CD48 and CD150 markers according to Kiel et al. (2005) to visualize the subtypes of the Sca-1 positive (LSK) cells with the HSCs, MPP and myeloid progenitor developmental potential. Figure 1A shows the representative sample wherein the immunophenotype of LK cells is shown in regenerating and normal bone marrow. The c-Kit expression level is significantly decreased (see also Supplementary Table S3) and the proportion of Sca-1 positive (LSK) cells is increased in the LK cells with low c-Kit expression level in regenerating bone marrow. The LSK cells are all CD48 positive and the fraction of CD150 positive cells is increased.

Figure 1B compares LK cell numbers and the ratio between Sca-1 negative (LS^neg^K) cells and LSK cells in untreated mice (CTRL) and mice examined 15 days after irradiation (6 Gy D15). In the regenerating bone marrow, the proportion of LSK cells was significantly increased and consequently the LS^neg^K/LSK ratio decreased. Figure 1B also shows the distribution of LSK cells in four CD48/CD150 subtypes in normal (CTRL) and regenerating (6 Gy D15) bone marrow. Because of the significantly altered c-Kit expression level of LK cells in regenerating bone marrow, we analyzed the CD48 and CD150 expression in LSK cells in the c-Kit^high^ or c-Kit^low^ gates separately.

We added an anti-CD71 antibody to the antibody cocktail used to stain analyzed bone marrow samples. Unexpectedly, there was a very high frequency of CD71 positive LSK cells in the regenerating bone marrow, which was in striking contrast to LSK cells in the normal bone marrow (Figure 1C). Since the CD71 marker corresponds to the transferrin receptor 1, which is highly expressed in erythroid cells and also induced by a low iron-body state, we functionally tested the possibility that increased erythropoietin stimulation due to post-irradiation anemia (see Supplementary Figure S2B) or a relative iron deficiency due to intensive erythropoiesis induced CD71 expression in LK cells in regenerating bone marrow. In order to differentiate these possibilities, we prevented the development of anemia by repeated transfusions of red blood cells. However, CD71 expression remained high in LSK cells in regenerating bone marrow and was equal in LSK and LS^neg^K cells (Figure 1C). In non-irradiated mice, we tested, with a negative outcome, whether CD71 expression in LSK cells would respond to administration of erythropoietin (EPO) or induction of a state of iron deficiency (Figure 1D). Further, we functionally tested LK cells with different CD71 expression, sorted either from normal or regenerating bone marrow, by *in vitro* clonogenic assays. The capacity to form clones of hematopoietic cells declined with the increasing CD71 expression, both in the cells sorted from normal or regenerating bone marrow (Supplementary Figure S3).

As the expression of CD71 in LSK cells was quite unusual in regenerating bone marrow, we plotted the CD71 expression level against that of the Sca-1 in LK cells from normal and regenerating bone marrow. The Sca-1/CD71 expression profile of LK cells, highly conserved in normal bone marrow, was significantly altered in regenerating bone marrow and presented as irregular cell clusters (Figure 1E). Occurrence of the clusters was not affected by red blood cell transfusions and polycythemia (Figure 1E).

These results confirm the previously reported decreased c-Kit expression, increased Sca-1 expression and increased CD150 expression in immature cells in post-irradiation bone marrow (Simonnet et al., 2009). They are novel in showing the virtual absence of CD48-negative LK cells, the expression of CD71 in LSK cells, and the clusters of LK cells with highly variable Sca-1/CD71 expression profiles. They demonstrate that the altered immunophenotype of LK cells is not induced by erythropoietin stimulation.

### LSK Cells in Regenerating Bone Marrow Are Similar to Granulocyte-Macrophage and Erythroid Progenitors

**FIGURE 2:**
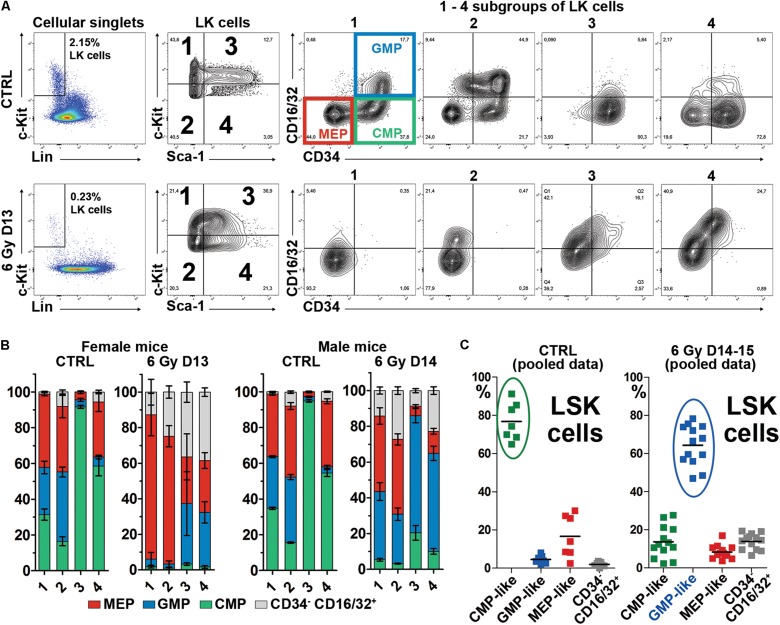
The CD34^+^CD16/32^–^ cells are significantly reduced in regenerating bone marrow and LSK cells express CD16/32. **(A)** Example of the CD34/CD16/32 expression profile in c-Kit^high^ – c-Kit^low^ and Sca-1^neg^ – Sca-1^+^ (subgroups 1, 2, 3, 4) LK cells. CTRL – an untreated mouse; 6 Gy D13 – a mouse irradiated with 6 Gy before 13 days. The CD34/CD16/32 immunophenotypes characteristic for the common myeloid progenitors (CMP), granulocyte-macrophage progenitors (GMP) and megakaryocyte-erythroid progenitors (MEP) shown in the subgroup 1 in the CTRL mouse were applied on cells in other subgroups of LK cells (2,3,4) and on the LK cells in the irradiated (6 Gy D13) mouse. **(B)** Proportion of cells with the MEP, GMP, CMP and CD34^–^/CD16-32^+^ immunophenotype profiles in 1, 2, 3, and 4 subgroups (see **A)** of LK cells in five untreated female mice (CTRL) and two female mice examined 13 days after irradiation (6 Gy D13), and four untreated male mice (CTRL) and six male mice examined 14 days after irradiation (6 Gy D14). **(C)** LSK cells were analyzed for the CD34/CD16-32 immunophenotype in 7 untreated mice (CTRL; from five independent experiments) and 13 irradiated mice examined 14–15 days after irradiation (6 Gy D14-15; in four independent experiments). All the mice were males. Cells immuphenotypically similar to CMP, GMP or MEP cells have the suffix “-like” because of the Sca-1 positivity. The increased expression of CD16/32 in LSK cells in regenerating bone marrow changed their major immunophenotype from the CMP-like to the GMP-like.

The unexpected finding of highly CD71 positive LSK cells in regenerating bone marrow prompted us to further explore LSK cells in regenerating bone marrow. We suspected that these cells could be developmentally restricted myeloid progenitors, originally Sca-1 negative, which had started re-expressing Sca-1. The myeloid Sca-1 negative progenitors can be further differentiated by means of their CD34 and CD16/32 (FcγRIII/II) expression profiles into the common myeloid progenitors (CMPs; CD34^+^CD16/32^–^), granulocyte-macrophage progenitors (GMPs; CD34^+^CD16/32^+^) and megakaryocyte-erythroid progenitors (MEPs; CD34^–^CD16/32^–^) (Akashi et al., 2000). As we became suspicious that LSK cells in regenerating bone marrow are myeloid progenitor cells that re-expressed Sca-1, we applied the CD34 and CD16/32 marker analysis on all LK cells in normal and regenerating bone marrow. We examined LK cells divided into four subsets: c-Kit^high^ – c-Kit^low^ and Sca-1 negative – Sca-1 positive (see Figure 2A, subgroups 1,2,3,4 of LK cells). Figure 2A shows examples of the CD34/CD16-32 expression profile in the four subsets of LK cells in normal (CTRL) and regenerating (6 Gy D13) bone marrow. Figure 2B shows the distribution of cells with the four CD34/CD16-32 immunophenotypes in female and male mice either normal (CTRL) or examined 13 days (6 Gy D13) or 14 days (6 Gy D14) after irradiation. The most striking difference between the LSK cells from normal and regenerating bone marrow is in the expression of CD16/32. LSK cells in normal bone marrow are uniformly CD16/32 negative, while regenerating bone marrow LSK cells are mostly CD16/32 positive and became GMP-like (Figure 2C).

**FIGURE 3:**
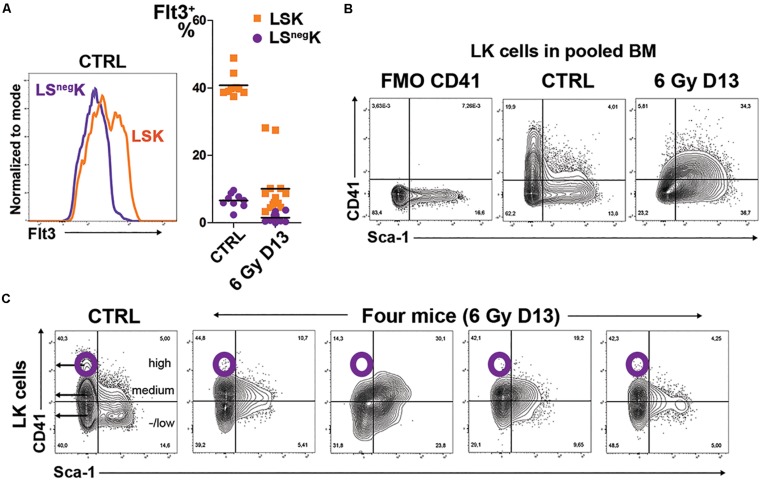
LSK cells in regenerating bone marrow have diminished expression of Flt3 and altered expression of CD41. **(A)** Flt3 expression in LS^neg^K and LSK cells in normal bone marrow (CTRL) and the percentage of Flt3 positive cells in LS^neg^K and LSK cells in 9 untreated mice (CTRL; data pooled from four independent experiments) and 13 mice irradiated with 6 Gy (6 Gy D13; data pooled from three independent experiments). All the mice were males. **(B)** CD41 expression in LK cells compared in samples of bone marrow pooled from two normal (CTRL) or three irradiated (6 Gy D13) mice. FMO CD41 is an aliquot of normal bone marrow stained with omission of anti-CD41 antibody. **(C)** CD41 expression in LK cells of one normal (CTRL) mouse and four mice examined 13 days after irradiation (6 Gy D13).

We compared also the expression of Flt3 (CD135/Flk2), which marks the lymphoid-biased MPPs (Buza-Vidas et al., 2011), in LK cells in normal and regenerating bone marrow. Approximately 40% of the LSK cells in normal bone marrow expressed Flt3 while the LS^neg^K cells were homogenously Flt3^low^ (Figure 3A). In regenerating bone marrow, the Flt3 expression became low in LSK cells and similar to its expression in LS^neg^K cells in normal bone marrow (Figure 3A).

The embryonic EMPs are mostly CD41 (integrin αIIb) positive (McGrath et al., 2015). Therefore, we determined CD41 expression in LK cells in normal and regenerating bone marrow. In normal bone marrow, CD41 is highly expressed in a fraction of LS^neg^K cells (corresponding to CMPs; not shown). LK cells in regenerating bone marrow lack the CD41^high^ cells and only a part of LSK cells express CD41 at a medium level (Figures 3B,C).

We also determined the expression of CD201 (EPCR) and CD105 (endoglin) in LK cells of normal and regenerating bone marrow. The expression of EPCR was decreased in regenerating bone marrow while the expression of endoglin was increased and correlated with the expression of CD71 (Supplementary Figure S4).

These results show that the LK cells in regenerating bone marrow are predominatly activated erythro-myeloid progenitors.

### Gene Expression Analysis Revealed Strongly Activated Erythroid Program Both in LSK and LS^neg^K Cells in Regenerating Bone Marrow

**FIGURE 4:**
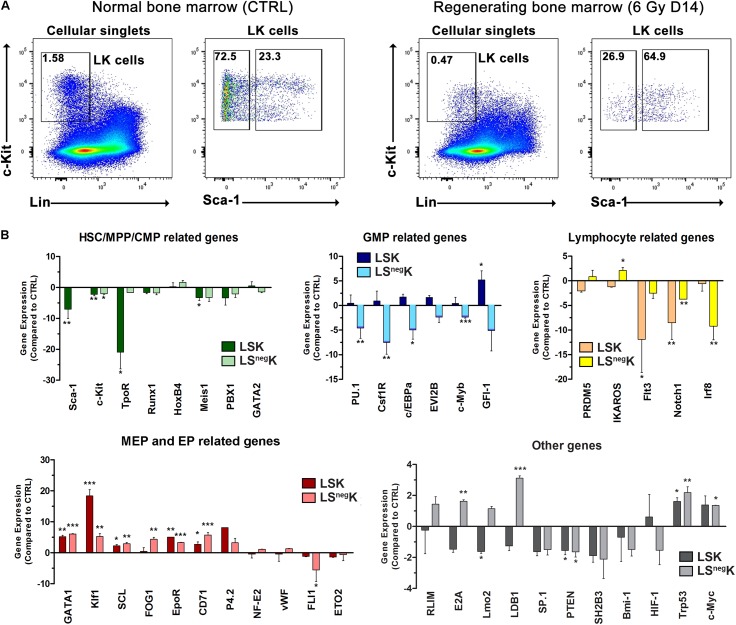
Expression of genes is significantly altered in LK cells in regenerating bone marrow. **(A)** Gating strategy used to sort LSK and LS^neg^K cells from normal (CTRL) or regenerating bone marrow collected 14 days after irradiation. **(B)** Inhibition or activation of gene expression in the LSK and LS^neg^K cells in regenerating bone marrow is shown as the difference against the expression in LSK or LS^neg^K cells in normal bone marrow. *P < 0.05, **P < 0.01, ***P < 0.001.

Because of the significantly altered immunophenotype of LK cells in regenerating bone marrow, we determined and compared the expression of genes important in hematopoiesis in the LSK and LS^neg^K cells sorted from normal and regenerating bone marrow (Figure 4A). We observed strongly upregulated expression of the *Gata1 and Klf1* genes and also of some other genes related to the erythroid developmental pathway, not only in LS^neg^K cells but also in LSK cells from regenerating bone marrow (Figure 4B). Moreover, the genes related to the granulocyte-macrophage developmental pathway (*PU.1, Csf1R, c/EBPa)* were low in LS^neg^K cells but appeared higher in the LSK cells in regenerating bone marrow (Figure 4B). The *Sca-1* gene, the gene for thrombopoietin receptor (TpoR; Mpl) and the *Meis1* gene, strongly expressed in normal LSK cells, were significantly decreased in LSK cells from regenerating bone marrow. Expression of the genes relating to lymphopoiesis (*Flt3, Irf8, Notch1*) was low in regenerating bone marrow (Figure 4B). Supplementary Figure S5A shows the expression levels of the examined genes in regenerating bone marrow compared to that of a reference housekeeping gene. We interpret these results as reflecting the significantly increased abundance of developmentally very late myeloid progenitors in LK cells in regenerating bone marrow, and the low abundance of HSCs, MPPs, CMPs, and also of the progenitors for the lymphoid developmental lineage.

Irradiated mice became anemic in the second week after irradiation (Supplementary Figure S2B). However, resolution of the anemia by transfusions of red blood cells and resulting polycythemia did not suppress the erythroid mRNAs in LK cells in mice with regenerating bone marrow (Supplementary Figure S5B).

### LK Cells in Regenerating Bone Marrow Significantly Differ From LK Cells in the Expanding Hematopoiesis in Fetal Liver and Early Postnatal Bone Marrow

**FIGURE 5:**
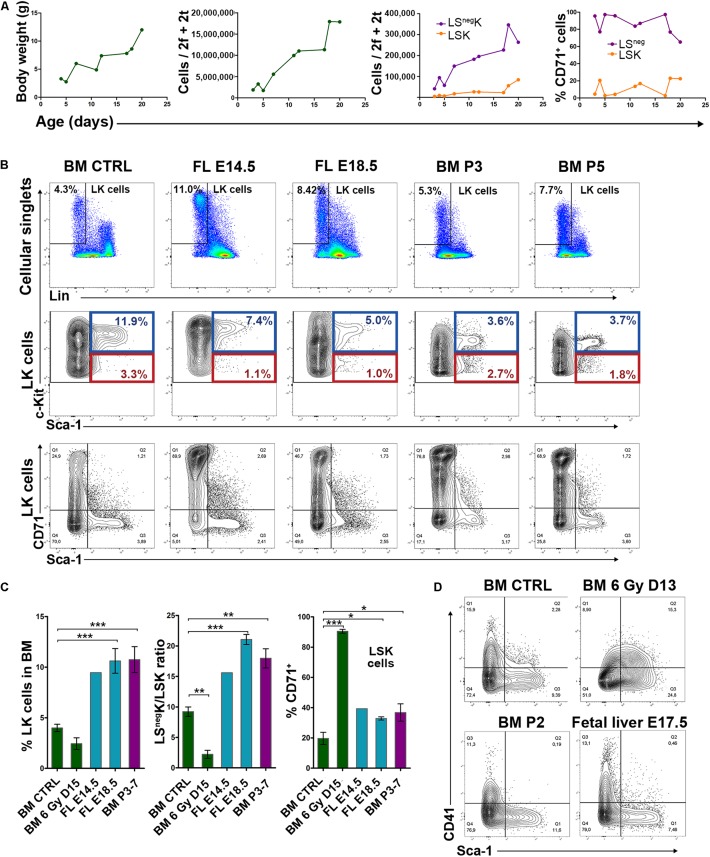
Expanding hematopoiesis in fetal liver and postnatal bone marrow compared to normal adult and regenerating bone marrow. **(A)** Body weight, bone marrow cellularity, LSK and LS^neg^K cells and the CD71 expression in LSK and LS^neg^K cells in postnatal mice. f – femur; t – tibia. **(B)** Examples of the immunophenotype of LK cells in adult bone marrow (Adult BM), fetal liver (FL-E14.5 or 18.5) and postnatal bone marrow (BM-P3 or P5). E – embryonic day; P – postnatal day. **(C)** Percentage of LK cells, LS^neg^K/LSK cell ratio and percentage of CD71^+^ LSK cells in normal adult BM (BM – CTRL; 5 untreated male mice from three independent experiments), regenerating adult BM (6 Gy BM – D15; 8 adult male mice examined 15 days after the irradiation with 6 Gy from three independent experiments), in FL (FL – E14.5 and E18.5; 3 and 4 mice) and postnatal BM (BM – P3-7; 4 mice examined 3–7 days postpartum). *P < 0.05, **P < 0.01, ***P < 0.001. **(D)**, Sca-1/CD41 expression profile in adult BM (BM CTRL), regenerating adult BM collected 13 days after irradiation (BM 6 Gy D13), BM collected on postnatal day 2 (BM P2) and fetal liver (FL – E17.5).

Regenerating bone marrow mimics the hematopoiesis in the fetal liver and early postnatal bone marrow by the rapid expansion of populations of immature cells accompanied by rapidly increasing production of mature blood cells. Therefore, we interrogated whether the vigorous expansion of regenerating bone marrow is similar in some aspects to the hematopoiesis in the fetal liver and the early postnatal bone marrow. Immunophenotyping of LK cells from the normal adult bone marrow, fetal liver and early postnatal bone marrow revealed significant differences from the LK cells in regenerating adult bone marrow (Figure 5B to be compared with Figure 1A). LK cells in the fetal liver and postnatal bone marrow were more frequent than in the adult and regenerating bone marrow (Figure 5C), exhibited high c-Kit expression (Figure 5B) and elevated LS^neg^K/LSK ratio (Figure 5C). The regenerating bone marrow lacked the CD41^high^ LK cells which occur in the LS^neg^K cells in the normal adult and postnatal bone marrow and also in the fetal liver (Figure 5D). CD41 is highly expressed in CMPs in normal bone marrow (not shown). The cells with the immunophenotype of CMPs are significantly suppressed in regenerating bone marrow (see Figure 2B) and the altered expression of CD41 thus correlates with this change in the composition of LK cells.

### Regenerating Bone Marrow Has a Slightly Decreased Capacity to Form *in vitro* Colonies in Semisolid Cultures

**FIGURE 6:**
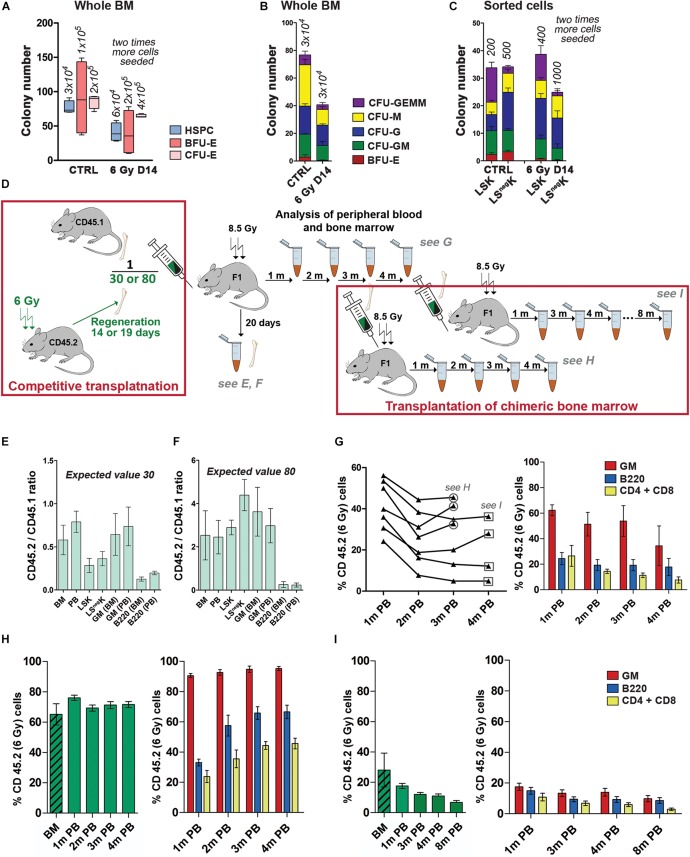
Developmental potential of LK cells from regenerating bone marrow in cell cultures and after transplantation. **(A–C)** Total number of colonies of hematopoietic cells obtained in *in vitro* cultures of normal (CTRL) or regenerating (6 Gy D14) whole bone marrow or sorted LSK or LS^neg^K cells. Results are pooled from three independent experiments. **(A)** Three types of culture media (from STEMCELL Technologies; Canada) were used. HSPC (Hematopoietic Stem and Progenitor Cells) is for the colonies cultured in the medium GF M3434 supporting growth of CFU-GEMM, CFU-GM, CFU-M, CFU-G and BFU-E cells; BFU-E is for the colonies cultured in the medium SF M3436; CFU-E is for cell clusters cultured in the medium M3334. Numbers of bone marrow cells seeded per dish are indicated in italics. **(B)** The “HSPC” colonies cultured in the GF M3434 medium were differentiated into various colony types. **(C)** LSK and LS^neg^K cells sorted from normal (CTRL) or regenerating (6 Gy D14) bone marrow were cultured in the medium GF M3434. Numbers of cells seeded per dish are indicated in italics. **(D)** Design of the experiments studying the transplantability and developmental potential of regenerating bone marrow. **(E,F)** The capacity of regenerating (CD45.2) and normal (CD45.1) bone marrow to restore blood cell production 20 days after transplantation (a short-term hematopoiesis repopulation) was tested. 30 **(E)** or 80 **(F)** fold more CD45.2 bone marrow cells, collected 19 days in **(E)** and 14 days in **(F)** after irradiation, were mixed with CD45.1 normal bone marrow cells and co-transplanted to lethally irradiated dual CD45.1/CD45.2 F1 (F1) recipient mice. CD45.2 and CD45.1 cells were determined in various types of nucleated blood cells (PB) and in bone marrow cells (BM; LSK, LS^neg^K) 20 days after transplantation. The CD45.2/CD451 ratio in the various types of cells is shown. **(G)** Regenerating (CD45.2; collected 14 days after irradiation of mice at 6 Gy) and normal (CD45.1) bone marrow were mixed in an 80: 1 ratio and transplanted (17.5 × 10^6^ per recipient mouse) to lethally (8.5 Gy) irradiated F1 mice. The percentage of CD45.2 nucleated blood cells, originating from regenerating bone marrow, was determined after 1, 2, and 3 months in seven individual mice and in four mice after 4 months. The percentage of CD45.2 Gr-1/Mac-1(GM), B220 and CD4 + CD8 blood cells is shown in the column diagram. **(H)** The CD45.2/CD45.1 chimeric bone marrow pooled from three mice sacrificed 3 months after transplantation (see panel **G)** was analyzed for the presence of CD45.2 cells (the hatched green column) and re-transplanted to secondary lethally irradiated F1 mice. Peripheral blood of the secondary transplanted mice was examined after 1, 2, 3, and 4 months similarly as after the first transplantation (see the empty green columns for all nucleated CD45.2 cells and the red, blue and yellow columns for the GM, B220 and CD4 + CD8 cells). **(I)** The CD45.2/CD45.1 chimeric bone marrow pooled from four mice sacrificed 4 months after transplantation of the mixture of CD45.2 and CD45.1 bone marrow cells (see panel **G)** was similarly treated as that of the three mice sacrificed 1 month earlier (see panel **H)**. Peripheral blood of the secondary transplanted mice was examined for the presence of CD45.2 cells for up to 8 months after transplantation.

The colony-forming potential of hematopoietic cells from regenerating bone marrow was determined in a series of *in vitro* experiments using three types of commercial culture media. Whole bone marrow cells or FACS-sorted LSK and LS^neg^K cells were plated into semisolid media which supported growth of either the erythroid progenitors (BFU-E and CFU-E) or a wide spectrum of the myeloid progenitors marked here as HSPCs (Hematopoietic Stem and Progenitor Cells) which included CFU-GEMM, CFU-GM, CFU-G, CFU-M and BFU-E progenitor cells. The results revealed the presence of various types of myeloid progenitor cells, both in whole normal and regenerating bone marrow, and also in sorted LS^neg^K and LSK cells (Figures 6A–C; Supplementary Figure S3). The capacity of LK cells from regenerating bone marrow to generate colonies of blood cell precursors was approximately a half of that of the LK cells from normal bone marrow.

### LK Cells in Regenerating Bone Marrow Have a Very Low Transplantation Potential

Expanding hematopoiesis in the fetal liver is very potent in transplantation and fetal liver cells outcompete bone marrow cells in co-transplantation assays (Rebel et al., 1996; Bowie et al., 2007; Copley et al., 2013). On the other hand, bone marrow collected 13 days after irradiation of mice with a dose of 5.5 Gy failed to produce blood cells when competitively transplanted with normal bone marrow (Harrison and Astle, 1982). This prompted us to perform a series of experiments which compared the transplantation power of the regenerating bone marrow to that of normal bone marrow. Preliminary experiments showed that regenerating bone marrow cells should be transplanted in a significant excess to the competing normal bone marrow cells in order to obtain comparable production of blood cells in both branches of resulting chimeric hematopoiesis. We then performed a series of experiments in which the transplantation and developmental potential of regenerating bone marrow was compared to those of normal bone marrow. The experimental design of these experiments is shown in Figure 6D.

In two experiments, we tested the short-term repopulating potential of co-transplanted regenerating and normal bone marrow. Cells from regenerating bone marrow (CD45.2) were given in a 30–80 excess to the cells from normal bone marrow (CD45.1). The proportion of CD45.2 and CD45.1 cells were determined in blood and bone marrow 20 days after transplantation (Figures 6E,F). The capacity of regenerating bone marrow to produce bone marrow and mature blood cells was only ∼ 2% of the capacity of normal bone marrow. Regenerating bone marrow also produced less B-cells (B220) than granulocytes and macrophages (GM; Figures 6E,F).

We then compared the long-term reconstitution of damaged hematopoiesis in mice transplanted with a mixture of regenerating and normal bone marrow cells and employed three experimental settings in these experiments.

First, we co-transplanted bone marrow collected from mice 14 days after irradiation (CD45.2) with normal bone marrow (CD45.1) at an 80:1 ratio. The peripheral blood of the recipient mice was analyzed for presence of CD45.2 nucleated blood cells for 4 months (Figure 6G). The 80-fold excess of transplanted regenerating bone marrow resulted in production of a half of blood cells of the regenerating bone marrow origin in three out of seven transplanted mice after 3 months (Figure 6G). More granulocytes and macrophages (GM cells) were produced by transplanted regenerating bone marrow than B-cells (B220) and T-cells (CD4 + CD8) (Figure 6G). To confirm the capacity of regenerating bone marrow to support hematopoiesis in the long-term further, bone marrow of the three mice was pooled, examined for the frequency of CD45.2 cells, and transplanted to secondary recipient mice. The transplanted chimeric bone marrow contained ∼60% of CD45.2 cells (Figure 6H, the hatched column). The production of CD45.2 blood cells remained steady after the second transplantation for 4 months and was skewed for myeloid (GM) cells (Figure 6H). Four mice with the chimeric bone marrow containing approximately 25% of CD45.2 cells (see Figure 6I, the hatched column) were sacrificed 4 months after co-transplantation of regenerating (CD45.2) and normal (CD45.1) bone marrow cells (see Figure 6G) and their bone marrow was transplanted to secondary recipient mice. The production of CD45.2 blood cells was then followed for 8 months (Figure 6I). The percentage of CD45.2 cells in peripheral blood steadily declined but CD45.2 cells were still present in the peripheral blood of the secondary transplanted mice after 8 months (Figure 6I). These experiments revealed the occurrence of transplantable cells with the long-term repopulating capacity constituted ∼1% of their occurrence in normal bone marrow since approximately hundred times more regenerating cells were needed to establish a 50: 50 chimeric hematopoiesis in the co-transplantation experiments.

Second, we compared the long-term reconstituting potential of LSK cells from regenerating and normal bone marrow. The regenerating bone marrow (CD45.2) was collected either 14 days or 23 days after irradiation. Normal bone marrow was from CD45.1 mice. To reduce a pre-transplantation stress to LSK cells to be transplanted, we first determined the number of LSK cells in aliquots of the CD45.2 and CD45.1 bone marrow samples kept at 4°C during the flow cytometry analysis of their aliquots. The regenerating and normal bone marrow cells were then mixed in the ratio wherein equal number of CD45.2 and CD45.1 LSK cells were present. The cell mixture was transplanted and the percentage of CD45.2 and CD45.1 nucleated blood cells was determined in the peripheral blood of transplanted mice after 1 and 6 months. Results of both these experiments revealed a very low capacity of the LSK cells from regenerating bone marrow to reconstitute and support hematopoiesis after transplantation (Table 2).

Third, we compared the transplantation potential of LSK CD150^+^CD48^–^ cells sorted from normal and regenerating bone marrow (for gating used see Supplementary Figure S6). The regenerating bone marrow was collected from mice (CD45.1) irradiated before 20 days. An equal number of LSK CD150^+^CD48^–^ cells obtained from regenerating (CD45.1) and normal (CD45.2) bone marrow were co-transplanted. The resulting chimeric hematopoiesis was then examined in the peripheral blood of recipient mice after 1 and 4 months and in the bone marrow after 5 months (Table 3). The LSK CD150^+^CD48^–^ cells obtained from regenerating bone marrow had a significantly reduced transplantation potential.

All these experiments thus demonstrated that expanding regenerating bone marrow contains a very low number (1–2%) of the cells which can reconstitute damaged hematopoiesis after transplantation compared to steady-state adult bone marrow.

### The c-Kit Receptor – Stem Cell Factor Interaction Is Essential for Expansion of Myeloid Progenitors in Regenerating Hematopoiesis

**FIGURE 7:**
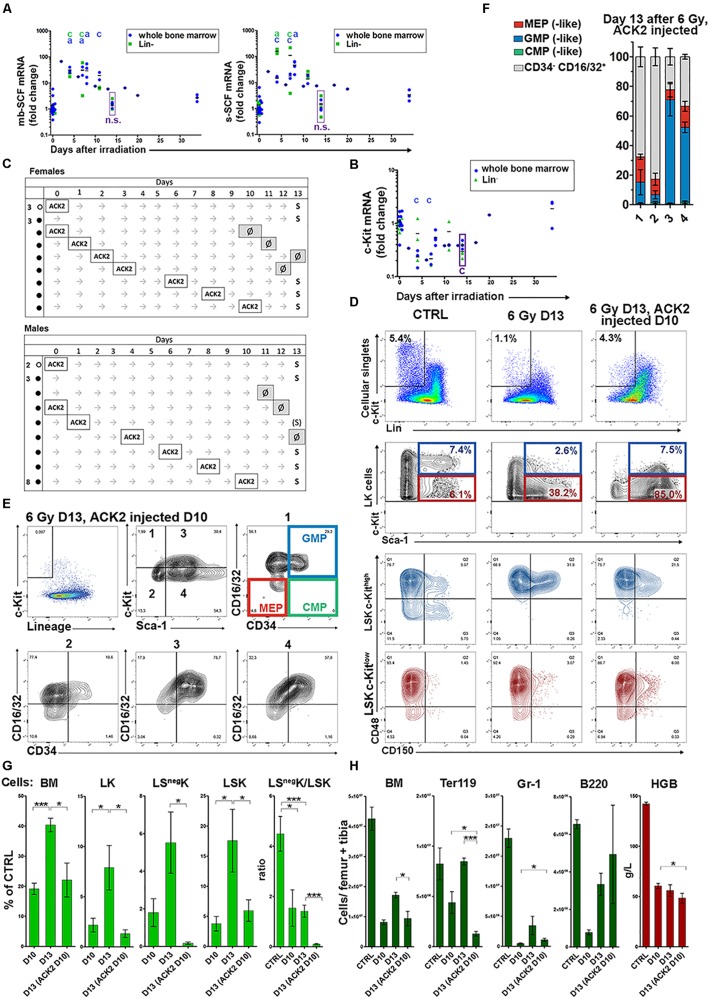
Abrogation of c-Kit receptor signaling reveals its role in bone marrow regeneration. **(A)** Stem cell factor (SCF) mRNAs level was significantly increased in total bone marrow cells or magnetically separated Lin^–^ cells of irradiated mice. mb – membrane-bound; s – soluble. **(B)** mRNA for c-Kit determined in all bone marrow cells and in magnetically separated lineage negative fraction of bone marrow cells (Lin^–^). The statistical significance between results from bone marrow of irradiated mice and untreated mice is indicated by letters a: *p* < 0.05, c: *p* < 0.005. **(C)** c-Kit blocking ACK2 antibody was lethal if given during first 4 days after irradiation (∙), except in one mouse. Normal mice (∘) and mice given ACK2 6, 8, and 10 days after irradiation all survived for 13 days. Mouse death is indicated with a Ø. **(D,E)** Representative immunophenotypes of Lin^–^c-Kit^+^ (LK) cells in bone marrow of untreated (CTRL), irradiated (6 Gy D13) and irradiated and given ACK2 10 days after irradiation (6 Gy D13, ACK2 injected D10) mice examined 13 days after irradiation. **(F,G)** The proportion of cells with the MEP-, GMP-, CMP-(like) and CD34^–^/CD16-32^+^ immunophenotype in four subtypes of LK (see panel **E**) in mice given ACK2 10 days after irradiation and examined 13 days after irradiation. For corresponding values in bone marrow of untreated mice (CTRL) and those of only 6 Gy-irradiated mice see Figure 2B. **(G)** Relative numbers of all bone marrow cells (BM) and of LK, LS^neg^K, LSK cells in six irradiated mice examined after 10 days or after13 days, and five irradiated mice given ACK2 3 days before examination on day 13. All mice were males, and the values from normal eight mice served as the reference 100% values. **(H)** Absolute number of total BM cells, Ter119^+^ cells, Gr-1^+^ cells and B220^+^ cells in these mice **(F)**. Also their hemoglobin concentration in the peripheral blood is shown. D10, D13 – days after irradiation with 6 Gy; HGB – hemoglobin concentration in blood. *P < 0.05, **P < 0.01, ***P < 0.001.

The low c-Kit expression level in LK cells in regenerating bone marrow was confusing since a high c-Kit expression level is a hallmark of hematopoietic stem and progenitor cells in the mouse (Okada et al., 1991; Osawa et al., 1996). We hypothesized that c-Kit might have been downregulated on the surface of LK cells by SCF as it occurs *in vitro* (Chen et al., 2016). Therefore, we determined the expression of the mRNAs for the membrane-bound and soluble forms of SCF. Both mRNAs were significantly upregulated after irradiation (Figure 7A). To resolve the discrepancy between the downregulation of c-Kit by SCF and the inherently decreased production of c-Kit in LK cells in regenerating bone marrow, we determined the mRNA for c-Kit in normal and regenerating bone marrow. The mRNA for c-Kit was low in regenerating bone marrow still 14 days after irradiation (Figure 7B) suggesting decreased production of c-Kit in LK cells. Therefore, we functionally tested the role of the c-Kit receptor mediated signaling in the development of myeloid progenitors in regenerating hematopoiesis by blocking c-Kit through the administration of a c-Kit-blocking antibody (ACK2) to mice. ACK2 effectively abrogated the c-Kit response to SCF for at least 3 days after a single dose of 0.5 mg (Supplementary Figures S7B,C). ACK2’s binding to c-Kit did not interfere with staining by fluorescently labeled anti-c-Kit antibodies (Supplementary Figure S7A) used for flow cytometry. In a series of experiments, ACK2 was given to mice following their irradiation, and also to untreated control mice. The mice were then examined 13 days after irradiation. Mice given ACK2 up to 4 days after irradiation did not survive, except for one which was in a moribund condition (see Figure 7C). The phenotype of LK cells, in their c-Kit^high^ and c-Kit^low^ fractions, in the mice given ACK2 1, 6, and 8 days after irradiation is shown in Supplementary Figure S8. The LK cells were predominantly Sca-1 and CD48 positive, CD150 negative and lacked CD71^high^ cells.

We then asked how much the reconstitution of damaged hematopoiesis still depended upon SCF/c-Kit signaling 10–13 days after irradiation, when the SCF mRNAs level started to steeply decline (see Figure 7A) but the mRNA for c-Kit (Figure 7B) and c-Kit expression on LK cells (Supplementary Table S3) was still low. All nine mice, eight males and one female, given ACK2 10 days after irradiation survived until day 13 (Figure 7C). In these mice, ACK2 treatment significantly inhibited the recovery of LK cells (Figure 7G), and particularly their LS^neg^K fraction (Figures 7D,E,G). LSK cells were also depressed after ACK2 administration (Figure 7G), but significantly less than LS^neg^K cells, as shown by the LS^neg^K/LSK cell ratio which was approximately 50-fold decreased in the ACK2-treated mice (Figure 7G). LK cells surviving ACK2 treatment in irradiated mice were almost exclusively positive for the CD16/32 expression (Figures 7E,F). The differentiated precursors of red blood cells (Ter119) and granulocytes (Gr-1) were significantly suppressed in ACK2 treated mice, in difference to B-cells (B220) which were not affected (Figure 7H).

These results reveal that the SCF/c-Kit signaling is essential for the development and population expansion of erythro-myeloid progenitors in regenerating bone marrow despite the decreased expression of c-Kit receptor in these cells.

## Discussion

In the present study, we provide a deep insight into the intensively regenerating bone marrow during its recovery from severe damage and compare it to the physiologically expanding embryonic/fetal liver and early postnatal hematopoiesis which also multiply their immature hematopoietic cells together with increasing production of mature blood cells, which are two competing tasks.

In the embryo, the transient primitive hematopoiesis starts in the yolk-sac and produces mainly large nucleated erythroblasts with the fetal type hemoglobin. This is followed by another transient hematopoiesis driven by erythro-myeloid progenitor cells which originate in the yolk sac and migrate to the newly established fetal liver. These two phases of the embryonic hematopoiesis precede the appearance of transplantable HSCs [Frame et al. (2013); McGrath et al. (2015); reviewed in Palis (2016) and Dzierzak and Bigas (2018)]. HSCs are then generated separately in the embryo proper by differentiation from the hemogenic endothelium of large arteries in the aorto-gonad-mesonephros region and from the vitelline arteries and in the placenta in a stepwise process (Rybtsov et al., 2014, 2016). HSCs migrate into fetal liver where they initiate production of all blood cells including the lymphocytes. The hierarchical structure of hematopoiesis with HSCs – MPPs and developmentally restricted progenitors is thus first established in the fetal liver. These HSCs are multipotent and have a high self-renewal and transplantation capacity connected with the activity of the Lin28b-let-7-Hmga2 axis (Copley et al., 2013). HSCs actively proliferate in the fetal liver and in bone marrow during the first 3 weeks of life in the mouse (Bowie et al., 2006). Afterward, many HSCs enter a dormant state but can still contribute to hematopoiesis (Sawai et al., 2016; Akinduro et al., 2018). The HSCs are induced to proliferate after bone marrow damage, infection, or sustained increased red blood cell production (Baldridge et al., 2010; Trumpp et al., 2010; Singh et al., 2018).

In our study, we targeted the transient period of bone marrow regeneration wherein the hematopoiesis, derived from a very small number of founder cells, is challenged with similar tasks as in the embryonic hematopoiesis: to concurrently produce blood cells in large amounts, expand the pools of progenitors and to reconstruct the hierarchical structure of hematopoiesis. We focused on all immature cells lacking lineage markers and expressing the c-Kit receptor (LK cells). This is in contrast to the study of Simonnet et al. (2009) which analyzed a subtype of LSK cells characterized by a high efflux of Hoechst 433342 dye (“side population” in flow cytometry; SP) in mice irradiated with either 3 or 6 Gy. They analyzed bone marrow in mice irradiated with 3 Gy after 2–14 days, and after 10 weeks in mice irradiated with 3 or 6 Gy. These authors found decreased c-Kit expression and increased Sca-1 expression in SP-LSK cells during first 4 days after the dose of 3 Gy, and increased expression of CD150 marker up to 10 weeks post irradiation. There was a significant deficit in Flk-2 (CD135) cells after 10 weeks and SP-LSK CD150^+^ cells showed reduced repopulating potential after transplantation. Our study thus principally differs from the study of Simonnet et al. (2009) in targeting all lineage-negative and c-Kit expressing cells (LK cells) in their c-Kit^low^ and c-Kit^high^ fractions, and also by targeting the transient regenerative phase characterized by the intensive production of mature blood cells and concurrent massive expansion of progenitor cells.

While studying Sca-1 expression in LK cells, we noticed Sca-1 expression in CD71-positive LK cells in regenerating bone marrow while the LSK cells in normal bone marrow were uniformly CD71 negative. CD71 is highly expressed in erythroid cells stimulated by erythropoietin and its expression in LSK cells in regenerating bone marrow might signal their activation toward the erythroid commitment. The functional *in vivo* tests did not support this hypothesis. Therefore, we hypothesized that the normal Sca-1 negative and CD71 expressing early erythroid progenitors, re-expressed the Sca-1 antigen in regenerating bone marrow as part of their activation.

All these findings prompted us to focus on LSK cells in regenerating bone marrow and their comparison with LS^neg^K cells. Since we became suspected that LSK cells in regenerating bone marrow are, in fact, the myeloid progenitor cells which re-expressed Sca-1, we applied the CD34 and CD16/32 markers, traditionally used only for analysis of LS^neg^K cells, also on the analysis of LSK cells. This analysis revealed several similarities between the LSK cells and LS^neg^K cells in regenerating bone marrow. LSK cells in regenerating bone marrow mostly lacked the Flt3 (CD135) positive cells which are the lymphoid-primed multipotent progenitors with down-regulated megakaryocyte-erythroid potential (Buza-Vidas et al., 2011). LSK cells in regenerating bone marrow were mostly CD16/32 positive and expressed CD71 at variable level. The expression of CD16/32 characterize the granulocyte-macrophage progenitors. The CD71 expression is linked to the erythroid developmental lineage.

The flow cytometry analysis of regenerating bone marrow thus uncovered expanded populations of cells with phenotypic markers of the erythroid and myeloid (granulocyte-macrophage) progenitors masked by expression of Sca-1 in part of them and by the expression of CD16/32 by majority of the cells. The CD16/32 expression makes these myeloid progenitors similar to the EMPs in the embryo (Frame et al., 2013; McGrath et al., 2015; Palis, 2016). However, there are significant differences between the EMPs and the regenerating myeloid progenitors since EMPs are c-Kit^high^, uniformly Sca-1 and CD150 negative, and all express CD41, while LK cells in regenerating hematopoiesis are c-Kit^low,^ partly Sca-1 and CD150 positive, and express CD41only in a small fraction of LSK cells.

The gene expression analysis revealed strongly activated erythroid program not only in LS^neg^K cells but also in LSK cells in regenerating bone marrow (Figure 4 and Supplementary Figure S5). Correspondingly, the number of erythrocytes reached its nadir on day 12 after irradiation and then began to increase (Supplementary Figure S2B). The blockade of c-Kit receptor suppressed the erythropoiesis in regenerating bone marrow significantly (Figure 7H). All these findings demonstrate a significant erythroid activity in immature LK cells in intensively regenerating hematopoiesis. This strong erythroid activity was not fully reflected in *in vitro* clonogenic cultures of cells from regenerating bone marrow (Figures 7B,C and Supplementary Figure S3). We hypothesize that the culture conditions in GF M3434 medium did not fully substitute for the support and stimulation provided to erythro-myeloid progenitors by the microenvironment in regenerating bone marrow, e.g., the membrane-bound SCF and macrophages known to participate in the erythroblast differentiation and maturation are missing in *in vitro* cultures and the conditions for the growth of erythroid cells are thus often suboptimal (Monette and Holden, 1982).

Analysis of the gene expression in LSK and LS^neg^K cells separated from normal and regenerating bone marrow further supported the erythro-myeloid character of LK cells, but revealed differences between their LSK and LS^neg^K subtypes. While LSK cells were both erythroid and granulocytic (myeloid) according to the enhanced gene expression linked to both these developmental lineages, the LS^neg^K cells had only the erythroid program enhanced. The suppression of erythropoietin stimulation by posttransfusion polycythemia did not inhibit the erythroid program in LK cells in regenerating hematopoiesis, nor did it suppress the enhanced expression of CD71. Peslak et al. (2012) showed that only Day-3 BFU-E and CFU-E erythroid progenitors were responsive to erythropoietin stimulation in regenerating bone marrow. Therefore, most of LK cells in regenerating bone marrow have a less advanced erythroid status than Day-3 BFU-E. Contrary to the apparent erythropoietin independence, the expansion of LK cells in regenerating bone marrow, and particularly of their LS^neg^K fraction, required stimulation by SCF mediated by the c-Kit receptor.

The LK cells in regenerating bone marrow markedly differed from the LK cells in the fetal liver and early postnatal bone marrow phenotypically and in their capacity to be transplanted. The expansion power of the fetal liver hematopoiesis can be transferred to adult mice with damaged hematopoiesis by transplantation (Copley et al., 2013). Therefore, we explored the capacity of regenerating bone marrow to be similarly transplanted. This revealed the most striking difference between the fetal liver hematopoietic cells and the regenerating bone marrow. The transplantation potential of regenerating bone marrow cells was very severely reduced and also deficient in production of the lymphoid cells.

The functional testing of cells from the intensively regenerating bone marrow by *in vitro* clonogenic assays showed their reduced capacity. We hypothesize that myeloid progenitors in regenerating bone marrow depend more on their direct interaction with the supporting tissue microenvironment, e.g., stimulation by the membrane bound SCF, than normal progenitors. Another possibility to understand this paradoxical phenomenon would be increased sensitivity of clonogenic cells from regenerating bone marrow toward *in vitro* handling and cultivation negative effects.

In the present paper, we describe significantly expanded populations of altered erythro-myeloid progenitor cells both in the Sca-1 positive and Sca-1 negative LK cells in regenerating bone marrow. The most significant feature of these cells, expressing c-Kit at a low level, is their extremely restricted capacity for reconstitution of damaged hematopoiesis after transplantation which contrasts with their massive performance in the production of blood cells *in situ* and their concurrent population expansion. Randall and Weissman (1998) described cells present in murine bone marrow phenotypically similar to HSCs (Sca-1^high^) lacking the c-Kit expression and the ability to reconstitute hematopoiesis in transplanted mice. Other reports found HSCs in c-Kit^low^ immature cells (Doi et al., 1997; Lian et al., 1999; Yang et al., 2002; Thorén et al., 2008; Grinenko et al., 2014). However, these cells were identified as having a high capacity to reconstitute damaged hematopoiesis after their transplantation which contrasts with the very low capacity for transplantation of the erythro-myeloid progenitors from regenerating bone marrow we describe here. Finally, the Ratajczak′s group identified very small pluripotent cells with embryonic features in murine bone marrow with the potential to regenerate damaged tissues (Kucia et al., 2006; Ratajczak et al., 2019). Our study could not identify the very few cells which were at the beginning of the bone marrow regeneration because of the significant initial damage to bone marrow.

However, and bearing the limitations of our study in mind, we provide compelling evidence that hematopoiesis in regenerating bone marrow contains expanded populations of activated erythro-myeloid progenitors which markedly outweigh the severely reduced populations of the short-term and long-term repopulating cells corresponding to MPPs and HSCs. We also find some previously unknown similarities between regenerating adult bone marrow and definitive embryonic hematopoiesis occurring before the emergence of hematopoietic stem cells.

## Data Availability Statement

The datasets generated for this study are available on request to the first author.

## Ethics Statement

The animal study was reviewed and approved by the Laboratory Animal Care and Use Committee of the First Faculty of Medicine, Charles University and the Ministry of Education, Youth and Sports of the Czechia.

## Author Contributions

KF collected and analyzed the data, and wrote the manuscript. C-LC, TH, MB, KS, PP, and FS collected and analyzed the data. NR analyzed the data. EN contributed to the experimental design, data interpretation, manuscript writing, and final approval of the manuscript.

## Conflict of Interest

The authors declare that the research was conducted in the absence of any commercial or financial relationships that could be construed as a potential conflict of interest.
