# Supplementary material for: Altered Erythro-Myeloid Progenitor Cells Are Highly Expanded in Intensively Regenerating Hematopoiesis
Source: Front Cell Dev Biol. 2020 Feb 25;8:98. doi: 10.3389/fcell.2020.00098 (PMC7051989; doi:10.3389/fcell.2020.00098)
Supplement: Supplementary file 1 [file Table_1.pdf]

Supplementary information

Supplementary Figure S1

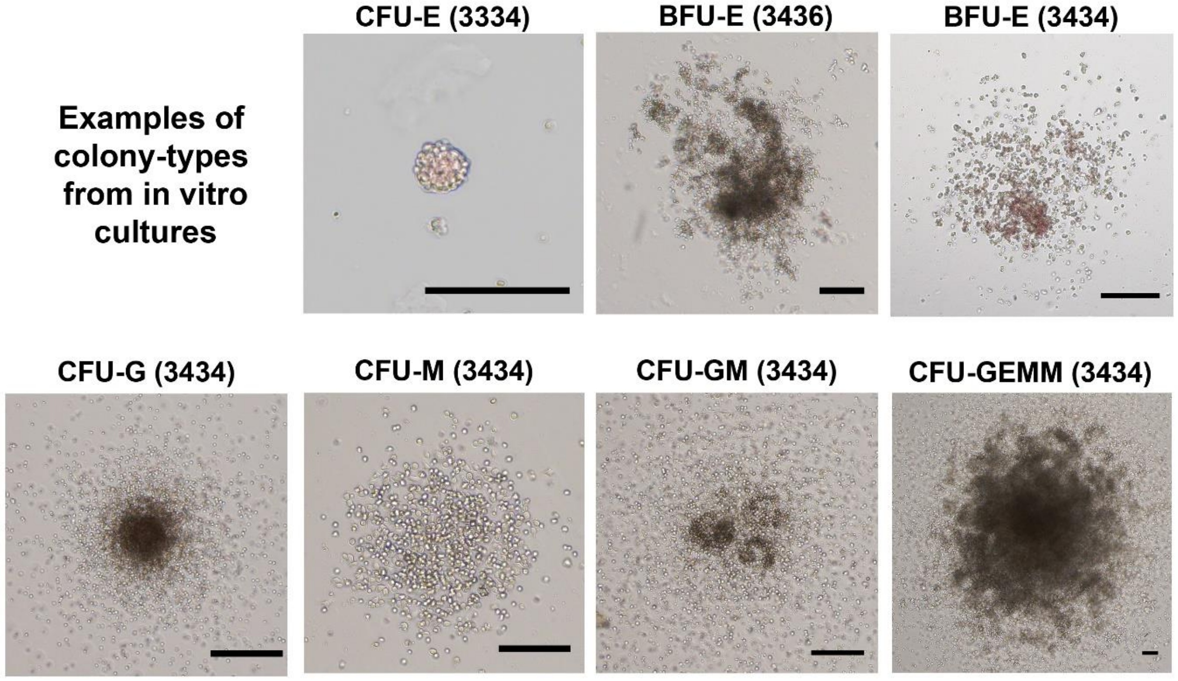

Representative images of colony-types in three semisolid media.

The images are from cultured whole bone marrow cells obtained from an untreated mouse. All media were from STEMCELL Technologies, Canada: M3334 medium for CFU-E clusters; SF M3436 medium for BFU-E colonies; GF M3434 medium for CFU-G, CFU-M, CFU-GM, BFU-E and CFU-GEMM colonies. The images were acquired by Olympus microscope (Olympus IX71) with color camera (Olympus DP74) and 4x UPlanFl (CFU-GEMM), 10x CPlanFl (all other) or 20x LCPlanFl (CFU-E) objectives. The scale bar is 100µm for each image.

Supplementary Figure S2

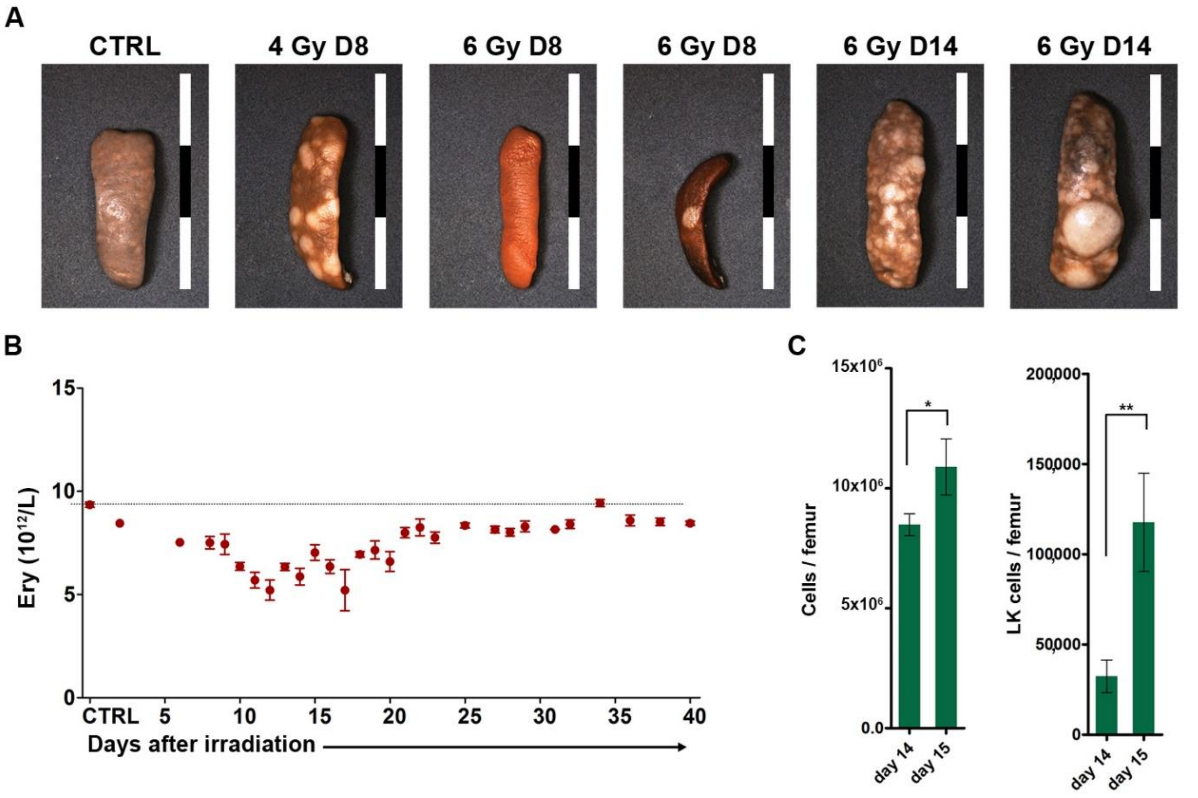

Endogenous spleen colonies, red blood cell numbers and expansion of hematopoiesis in bone marrow between days 14 and 15 after irradiation of mice with a dose of 6 Gy

A, Representative spleens of untreated (CTRL) and submyeloablatively irradiated mice; D – day after irradiation; each segment of the scale bar equals 5 mm. Two spleen are shown after irradiation of mice at 6 Gy for spleen collected either after 8 or 14 days.

B, Red blood cell (Ery) number achieves its nadir 12 days after irradiation of mice with 6 Gy. Data are from 52 untreated mice and 131 irradiated mice (both males).

C, Bone marrow cellularity and the number of immature $Lin^-c\text{-}Kit^+$ (LK) cells significantly increased between days 14 (16 male mice) and 15 (12 male mice) after irradiation. There were 28.255 ± 5.386 million of cells and 451,309 ± 153,982 LK cells in femur of untreated mice (mean ± SD; 127 male mice).

Supplementary Figure S3

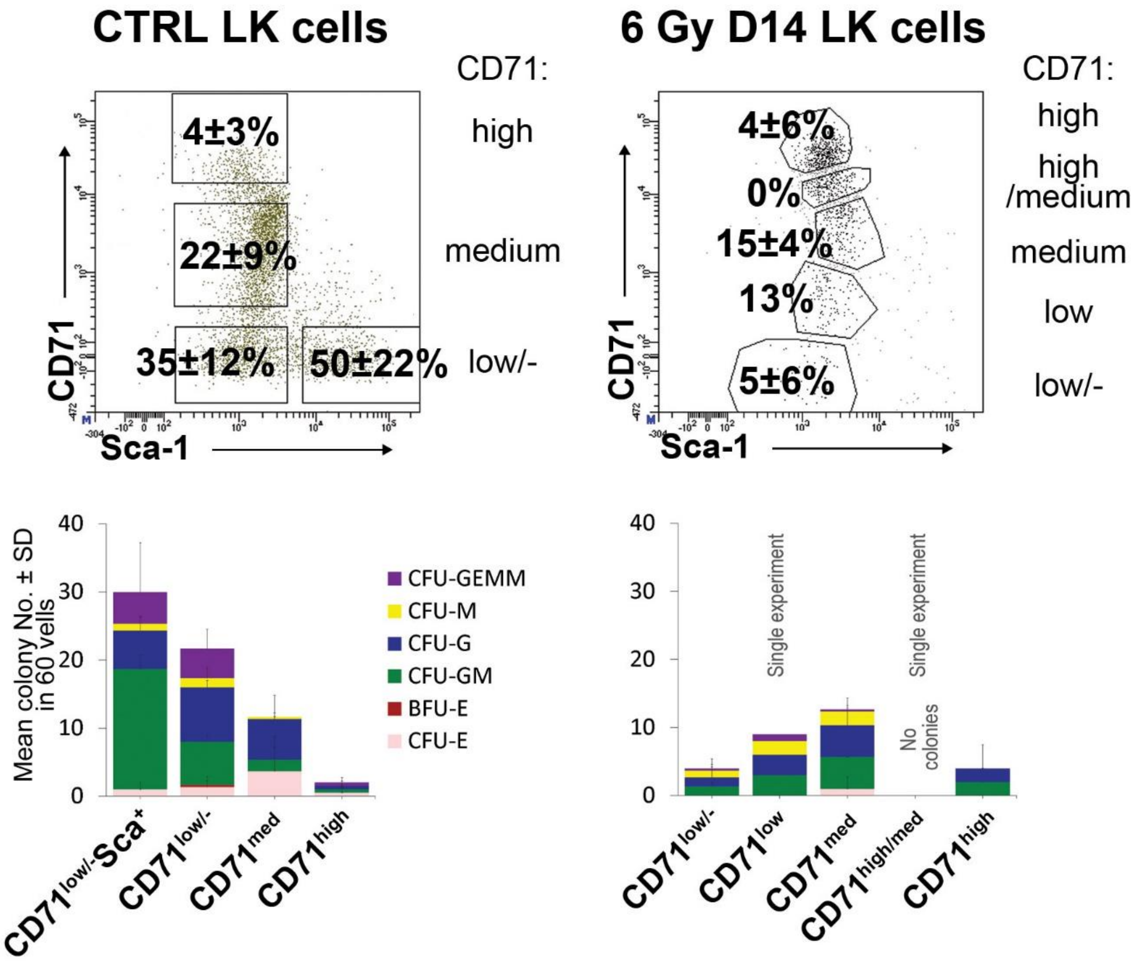

Functional analysis of LK cells with various expression of CD71 in normal and in regenerating bone marrow.

Single LK cells were sorted into the GF M3434 culture medium (STEMCELL Technologies, Canada) using a 96-wells plate. Sixty wells were used for cell cultures, and the remaining wells on the plate edge were filled with water to prevent the desiccation of medium. Positive wells containing hematopoietic cell colonies were determined after 10 days, and the colony types were established. The LK cells either from normal (CTRL) or regenerating bone marrow (6 Gy D14) were sorted based on Sca-1 and CD71 expression (see an example of the gates used). The plating efficiency is shown as the percentage of colony-positive wells (mean ± SD) for the cells from each gate. The number and the proportion of colony types are shown as the mean ± SD in the column graphs. Results are from three independent experiments, except CD71^{low} and CD71$^{high/medium}$ which were examined in a single experiment.

Supplementary Figure S4

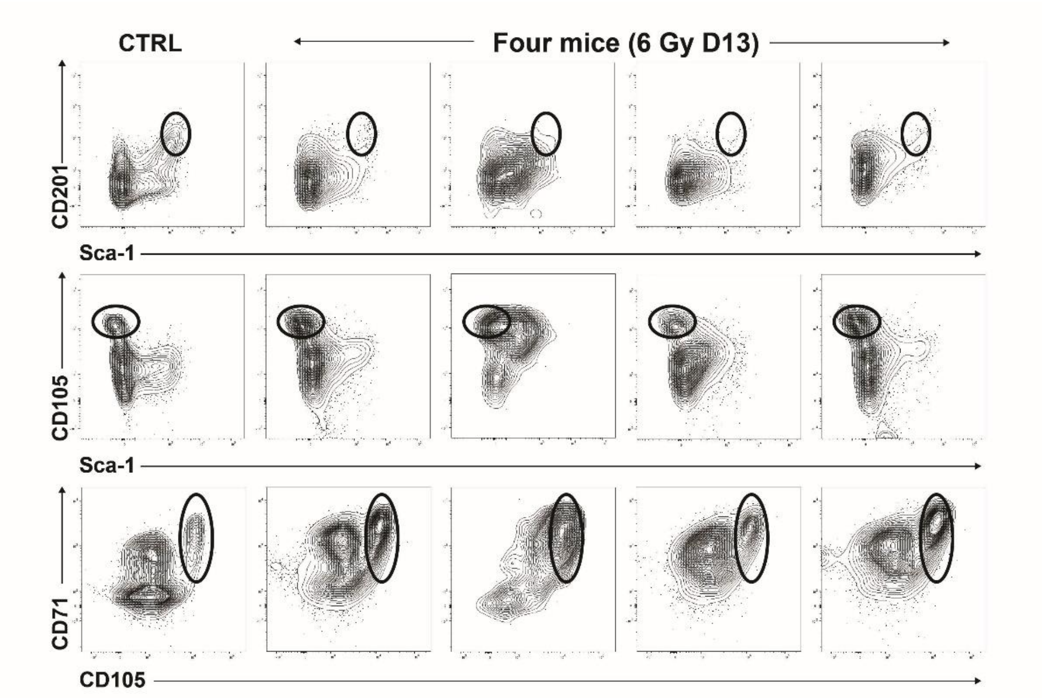

The expression of CD201 (EPCR) and CD105 (endoglin) in normal and regenerating bone marrow.

The representative CD201/Sca-1, CD105/Sca-1 and CD105/CD71 expression profiles in LK cells of normal bone marrow are shown (CTRL). The same expression profiles are shown in bone marrow collected from four mice examined 13 days after irradiation (6 Gy D13).

Supplementary Figure S5

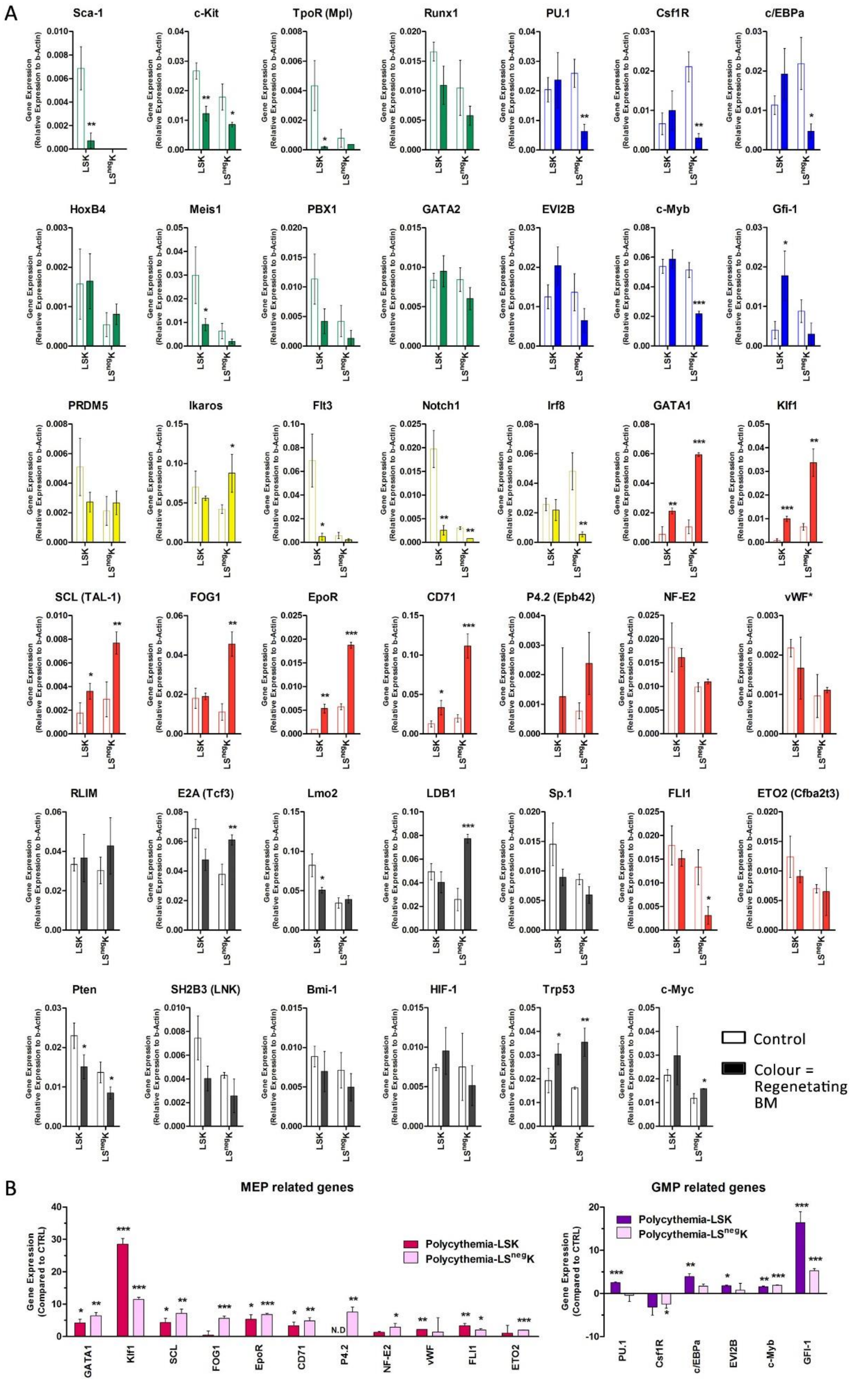

The expression level of studied genes compared to that of beta-actin reference gene and the effect polycythemia on the expression of MEP and GMP related genes

A, Expression of studied genes in LSK and $LS^{neg}K$ cells obtained either from normal bone marrow or bone marrow collected 14 days after irradiation of mice with a dose of 6 Gy. The expression level is compared to that of beta-actin (*bAct*). Results show mean ± SD from four biological replicates in the control group and six biological replicates in the 6 Gy-irradiated group. * $P<0.05$, ** $P<0.01$, *** $P<0.001$.

B, Activation or inhibition of genes associated with megakaryocyte-erythroid progenitors (MEP) or granulocyte-macrophage progenitors (GMP) in four irradiated mice (6 Gy) given transfusions of red blood cells which resulted in polycythemia. Bone marrow was collected on day 14 after irradiation, was pooled, and LSK and $LS^{neg}K$ cells were sorted for RNA isolation as shown in Figure 5A. These results correspond to those from mice with post-irradiation anemia (compare with Figure 4B).

Supplementary Figure S6

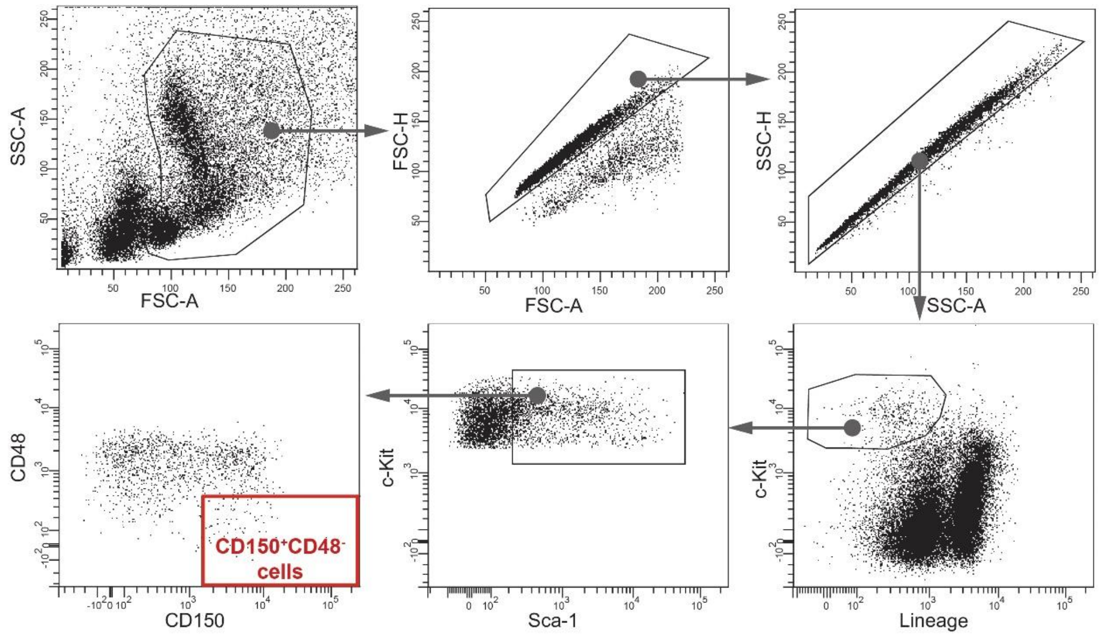

The gating used to separate LSK CD150^{+}CD48^{-} cells from either normal or regenerating bone marrow.

Supplementary Figure S7

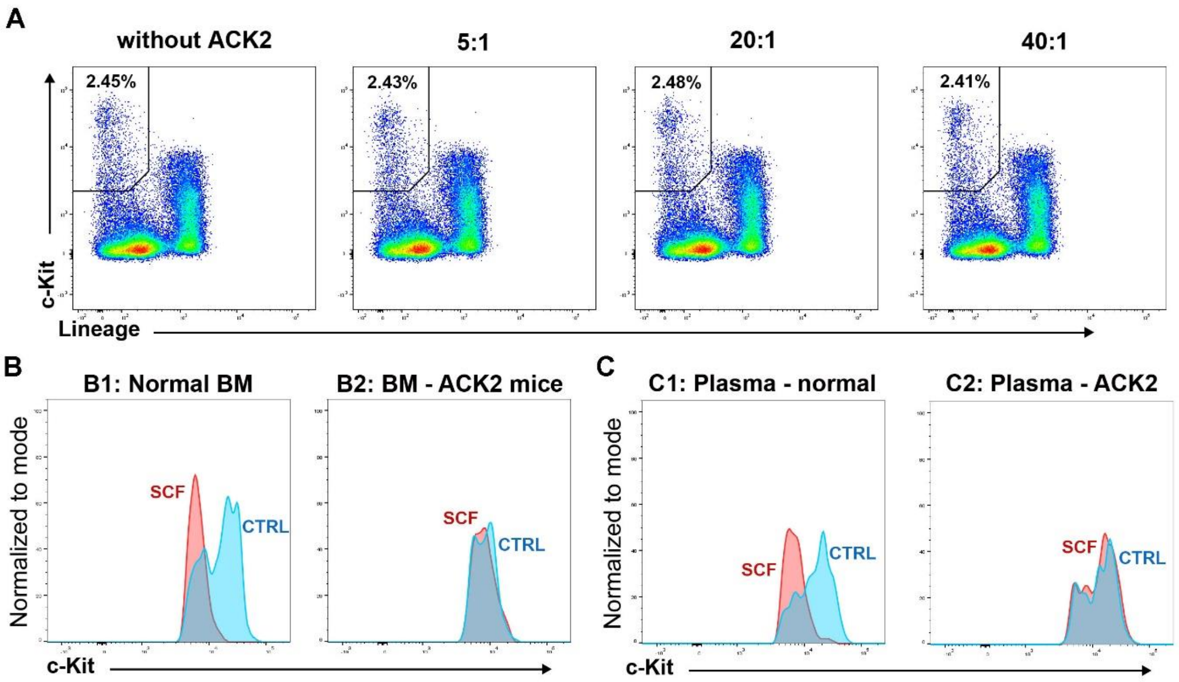

ACK2 bocks c-Kit efficiently for at least 3 days and does not interfere with c-Kit detection

A, Aliquots of ten million normal bone marrow cells in 100 µl PBS were incubated at 4°C for 20 minutes in the presence of zero - 0.125 - 0.5 – 1.0 µl of ACK2 antibody (2 mg/ml). Without washing, fluorescent-labeled antibodies against lineage markers (cocktail LIN; A700), Sca-1 antigen (SCA1; Pe-Cy7) and c-Kit (2B8; BV421) were added and the incubation continued for another 20 minutes. The ratio of ACK2/2B8 antibodies concentrations were 5:1, 20:1 and 40:1. Samples were then washed and the percentage of $Lin^{-}c\text{-}Kit^{+}$ cells compared by flow cytometry. The pre-incubation of bone marrow cells with ACK2 antibody did not affect c-Kit detection.

B, Bone marrow was collected into DMEM medium from normal mice (**B1**) and from mice given 0.5 mg of ACK2 antibody three days previously (**B2**). The bone marrow cells were split into two aliquots of ten million cells, each in 1 ml of DMEM. One sample (SCF) was incubated with 200 ng of SCF while the second sample (CTRL) was incubated without SCF (30 minutes at 37°C). After washing, cells were stained with fluorescent-labeled antibodies against lineage markers (cocktail LIN; A700), Sca-1 antigen (Sca1; Pe-Cy7) and c-Kit (2B8; BV421) and the c-Kit fluorescence intensity was compared. Normal bone marrow cells down-regulated $c\text{-}Kit^{+}$ receptors when exposed to SCF (**B1**) but the cells from ACK2-treated mice did not respond to SCF (**B2**).

C, Blood plasma was obtained from untreated mice and mice given 0.5 mg of ACK2 antibody three days before blood (plasma) collection. Normal bone marrow cells, in 200 µl of PBS were pre-incubated for 20 min at 4°C with 300 µl of plasma from either untreated mice (**C1**) or from ACK2-treated mice (**C2**). Both samples were then split into two aliquots and incubated for 30 minutes at 37°C in the presence (SCF; 300 ng per ml) or absence (CTRL) of SCF. After washing, cells were stained with fluorescently-labeled antibodies against lineage markers (cocktail Lin A700), Sca-1 antigen (Sca1 Pe-Cy7) and c-Kit (2B8; BV421), and c-Kit fluorescence intensity was compared. Pre-incubation with the plasma from

ACK2-treated mice (**C2**) abolished the SCF-induced down-regulation of c-Kit receptors which occurred in the cells pre-incubated with the plasma of untreated mice and exposed to SCF (**C1**).

Supplementary Figure S8

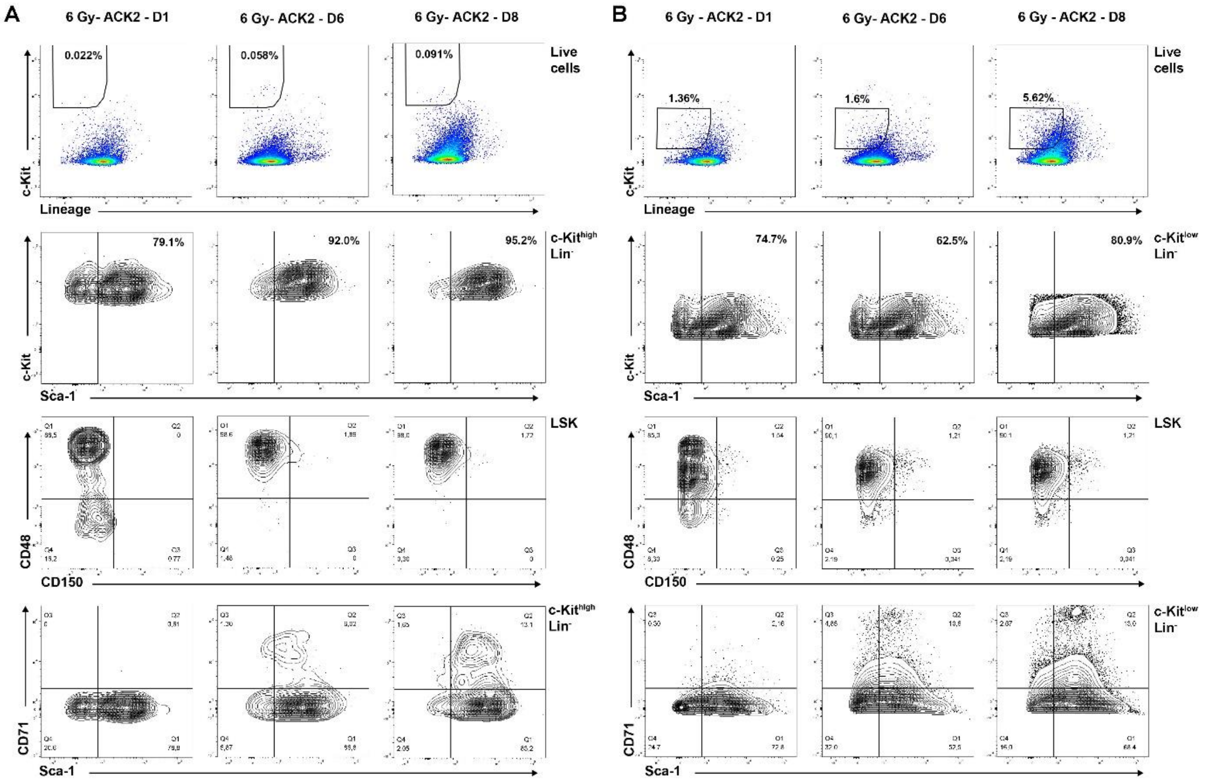

Administration of ACK2 antibody to mice irradiated with 6 Gy suppressed restoration of Lin$^-$c-Kit$^+$ (LK) cells and highlighted Sca-1$^+$ cells.

Bone marrow was analysed in three mice that survived for 13 days after irradiation (6 Gy) and the administration of 0.5 mg of ACK2 antibody 1, 6 or 8 days (D1, D6, D8) after irradiation. The mouse given ACK2 1 day after irradiation was in a moribund condition (17.6 g b.w., 1.4×10^6 cells per femur, 0.022% LK cells which lacked CD71$^+$ cells). Sca-1$^+$ cells were highly abundant in all the mice.

A, c-Kit^{high}. **B,** c-Kit^{low} LK cells.

For normal bone marrow (CTRL), see Figs 1-4 and Extended Data Fig. 3.

Supplementary Table S1

Reagents used in immunophenotyping of hematopoietic cells

Antibody	Clone	Producer	Fluorochrome
anti-Lineage cocktail of antibodies CD45R(B220)/CD3/Ly-6G(Ly-6C)/CD11b/Ter-119)		http://www.biolegend.com/	Alexa Fluor® 700
CD117 (c-kit)	2b8	http://www.biolegend.com/	Brilliant Violet 421™
Ly-6A/E (Sca-1)	D7	http://www.biolegend.com/	PerCP
Ly-6A/E (Sca-1)	E13-161.7	http://www.biolegend.com/	PE/Cy7
CD48	HM48-1	http://www.biolegend.com/	PE
CD48	HM48-1	http://www.biolegend.com/	FITC
CD150 (SLAM)	TC15-12F12.2	http://www.biolegend.com/	APC
CD150 (SLAM)	TC15-12F12.2	http://www.biolegend.com/	Brilliant Violet 605™
CD71	RI7217	http://www.biolegend.com/	FITC
CD71	RI7217	http://www.biolegend.com/	PE
CD45.2	104	http://www.biolegend.com/	PE/Cy7
CD41	MWReg30	http://www.biolegend.com/	APC
CD41	MWReg30	http://www.biolegend.com/	PE
CD45.1	A20	http://www.biolegend.com/	APC
CD45R/B220	RA3-6B2	http://www.biolegend.com/	FITC
CD4	53-6,7	http://www.biolegend.com/	PerCP
CD8	GK1.5	http://www.biolegend.com/	PerCP
Ly-6G(Gr-1)	RB6-8C5	http://www.biolegend.com/	Brilliant Violet 421™
CD11b	M1/70	http://www.biolegend.com/	Brilliant Violet 421™
TER-119/Erythroid Cells	TER119	http://www.biolegend.com/	PerCP
CD 127 IL7	93	http://www.biolegend.com/	Brilliant Violet 785™
CD 16/32	A7R34	http://www.biolegend.com/	Brilliant Violet 510™
CD 34	RAM34	http://www.ebioscience.com/	Biotin
CD 135	A2F10	http://www.biolegend.com/	APC

Streptavidin		http://www.biolegend.com/	PE/Cy7

Supplementary Table S2

Primers sequence used for qPCR

**HSC/ MPP/ CMP related Genes**		**Forward Sequence**	**Revers Sequence**	**NCBI accession #**
Sca-1	lymphocyte antigen 6 complex	GTTTGCTGATTCT TCTTGTGG	TAACTGCTGCCTC CTGAGTA	NM_001271416. 1
c-Kit	KIT proto-oncogene receptor tyrosine kinase	TTACGTGAACACA AAACCAG	CCAAATGGTGAC ACAGATAC	NM_001122733. 1
TpoR	myeloproliferative leukemia virus oncogene	CTGTATGCCTACC GAGGAG	TCTGGTTGAGGGA CACATT	NM_001122949. 2
Runx1	runt related transcription factor 1	TACTCGGCAGAAC TGAGAAA	TGGTAGGTGGCA ACTTGTG	NM_001111021. 2
HoxB4	homeobox B4	GGAGTTGGAGAA GGAGTTTCAC	AGCGGATCTTGGT GTTG	NM_010459.7
Meis1	Meis homeobox 1	CCTCTTCCCTCTC TTAGCAC	CCTTAACACTTGT ATGGCTTG	NM_010789.3
Pbx1	pre B cell leukemia homeobox 1	ATCACAGTCTCCC AGGTATC	GGAGTAGAGGGC GAGTTAG	NM_183355.3
Gata2	GATA binding protein 2	CAACACACCACCC GATAC	TTCTGAGCAGGAG CGAG	NM_008090.5

**GMP (granulocyte-macrophage progenitor) related genes**				
PU.1	spleen focus forming virus (SFFV) proviral integration oncogene	CTTCCCTTATCAA ACCTTGTCC	GAACTGGTAGAG GCGAAT	NM_011355.2
Csf1R	colony stimulating factor 1 receptor	GAGCATCTTTGAC TGCGT	GCCATTTGGTATC CATCCTT	NM_001037859. 2
c/EBPa	CCAAT/enhancer binding protein (C/EBP), alpha	TGTATCTGGTCTC TGTGTCC	GAGTCTCAGTTTG GCAAGAA	NM_007678.3
EVI2B	ecotropic viral integration site 2b	ATCAGCGGTCAAC AACT	AGAAGAGGTAGG AGCAGG	NM_001077496. 1
c-Myb	myeloblastosis oncogene	CTCCCACACCATT CAAACA	TCCACCTCCTGCT TGATTT	NM_001198914. 1
Gfi-1	growth factor independent 1	ACATCTGCTCATT CACTCG	CCTGTGTGCTTTC TGCTA	NM_010278.2

**Genes related to lymphopoiesis**				
Prdm5	PR domain containing 5	GGAGAAGCGAAT GCCTG	GAGATGGTGCCTC GTGA	NM_027547.2
Ikaros	IKAROS family zinc finger 1	GCGATGCCACAA CTACTT	CCTCTCTGCTCCT ATCTTGC	NM_001025597. 2
Flt3	FMS-like tyrosine kinase 3	AGTTCAGTTACAC CCGCC	GCAAAGCAAAGG AGGTCT	NM_010229.2

Notch1	notch 1	TGTGGCTTCCTTC TACTGCG	TTTGCCGTTGACA GGGTT	NM_008714.3
Irf8	interferon regulatory factor 8	TTACAATCAGGAG GTGGATGC	CGGTCAGTCACTT CTTCAAAAT	NM_001301811. 1

**MEPs (megakaryocyte-macrophage progenitor) related genes**				
Gata1	GATA binding protein 1	AGTGTGTGAACTG TGGAGCA	GAGTGTTGTAGTG GTCGTTT	NM_008089.2
EpoR	erythropoietin receptor	GAGCACCTATGAC CACCCA	CACTCCAGAATCC GCTGAA	NM_010149.3
P4.2	erythrocyte membrane protein band 4.2	CAAACGGGAGAG CAACC	ACCTGAAGCAAG AGTGAG	NM_013513.3
Klf1	Kruppel-like factor 1 (erythroid)	GGCGAACTTTGGC ACCTAA	ATAAGGCTTCTCT CCCGT	NM_010635.2
CD71	transferrin receptor	GGCTACCTGGGCT ATTGTAA	GGTGTCAGCAAA CTCTATG	NM_011638.4
Scl	T cell acute lymphocytic leukemia 1	TCAATGACCAGG AGGAGGAA	GGGAAGTGTGCTT GGGTGTT	NM_011527.3
Fog1	zinc finger protein, multitype 1	ATCCCCTGAGAGA GAAGAAC	CCAGAAGGCACC ACTTT	NM_009569.4
NF-E2	nuclear factor, erythroid derived 2	TGGAGAGATGGA ACTGACT	ATAAGATGGTGG GGGAAGG	NM_001302338. 1
vWF	Von Willebrand factor	CAGGTGTCCGTGA TACAGT	GCTCCGTGGATTT GTGAAG	NM_011708.4
Fli1	Friend leukemia integration 1	GGGGAGTTCAAA ATGACGG	GGATGTCTCTGTT GGATGTG	NM_008026.5
Eto2	CBFA2/RUNX1 translocation partner 3	CAGAAGAATGGA AGCACCT	GTTCCATCTTGGC ACGC	NM_009824.2

**Other genes**				
Rlim	ring finger protein, LIM domain interacting	ATGAAAGTGGCTC TCTGC	AAGTTTGTTGCCT TCTGTG	NM_011276.4
E2A (Tcf3)	transcription factor 3	GCAGCAGTGACC AGAACA	CTCCCAAAGGTGG CATAG	NM_001164147. 1
Lmo2	LIM domain only 2	CTACAAGCTGGG ACGGAA	TGTCGGAGTTGAT GAGAAGG	NM_008505.4
Ldb1	LIM domain binding 1	CTCCGACTCTGTG TGATACT	TCCGTTTCCTCCG TTTGCTG	NM_001113408. 2
Sp.1	trans-acting transcription factor 1	TAGTTCTCAAGGC CAGACG	AAGGATTTGTTGT TGCTGTG	NM_013672.2
Pten	phosphatase and tensin homolog	GGACCAGAGACA AAAAGGG	GGCAGACCACAA ACTGAG	NM_008960.2
SH2B3	SH2B adaptor protein 3	GGAGATGCCTGA CAACC	GCTCTGCCGCTAA GGAAA	NM_008507.4

Bmi-1	Bmi1 polycomb ring finger oncogene	GGAAGAGGTGAA TGATAAAAGG	CATGACGTCAATC TGGAAAG	NM_007552.4
Hif-1a	hypoxia inducible factor 1, alpha subunit	GCCTAACAGTCCC AGTGA	GAGCCAGCATCTC CAAATCTA	NM_001313919.1
Trp53	transformation related protein 53	TAGGTAGCGACTA CAGTTAG	GGATATCTTCTGG AGGAAGTAG	NM_011640.3
c-Myc	myelocytomatosis oncogene	CCTCCACAAGGA AGGACTAT	TCACGCAGGGCA AAAAAG	NM_010849.4
SCF	stem cell factor	GCCTTATACTGGA AGAAGAAAC	GATACGTCCACAA TTACACC	NM_013598.3
mb-SCF	membrane-bound stem cell factor	TCCCGAGAAAGG GAAAGC	CTGCCCTTGTAAG ACTTGACTG	NM_001347156.1
s-SCF	soluble stem cell factor	TTATGTTACCCCC TGTTGCAG	CTGCCCTTGTAAG ACTTGACTG	NM_013598.3

**Housekeeping genes**				
Actb	musculus actin, beta	AGACTTCGAGCA GGAGAT	ATGCCACAGGATT CCATAC	NM_007393.5
GAPDH	glyceraldehyde-3-phosphate dehydrogese	TGTTCCTACCCCC AATGT	GGGAGTTGCTGTT GAAGTC	NM_001289726.1

Supplementary Table S3

	IRR	N	LS^-K	LSK	LSK $CD150^-$ $CD48^+$	LSK $CD150^-$ $CD48^-$	LSK $CD150^+$ $CD48^-$	LSK $CD150^+$ $CD48^+$
**♂ CD45.2**	**CTRL**	4	72.64 ±1.66	78.94 ±2.88	64.61 ±1.92	75.87 ±3.03	95.52 ±3.53	78.96 ±3.17
	**Day 14**	4	42.36 ±0.88	52.76 ±3.44	38.74 ±2.53	31.01 ±1.05	44.10 ±5.72	51.35 ±4.80
	**t-test**		**t=16.08; P<0.0001**	**t=5.827; P=0.0011**	**t=8.140; P=0.0002**	**t=14.02; P<0.0001**	**t=7.644; P=0.0003**	**t=4.803; P=0.0030**
**♂ CD45.1**	**CTRL**	4	25.91 ±1.89	36.11 ±3.52	39.29 ±4.20	36.95 ±4.21	47.29 ±5.01	33.14 ±2.93
	**Day 15**	5	17.97 ±1.65	23.87 ±1.33	26.66 ±1.47	Not enough cells	27.39 ±2.29	24.31 ±1.00
	**t-test**		**t=3.170; P=0.0157**	**t=3.555; P=0.0093**	**t=3.120 P=0.0168**	**-**	**t=3.895 P=0.0059**	**t=3.140 P=0.0164**

c-Kit fluorescence intensity (MFI) is decreased in various types of the Lin^-Kit^+ (LK) cells in regenerating bone marrow.

The mean fluorescence intensity (MFI) is in thousands of arbitrary units. All compared data were acquired in same instrument run. Gating for LK cells was set to cover approximately 1.5-2.0 % of total bone marrow cells in normal mice.
